# Specific Human ATR and ATM Inhibitors Modulate Single Strand DNA Formation in *Leishmania major* Exposed to Oxidative Agent

**DOI:** 10.3389/fcimb.2021.802613

**Published:** 2022-01-04

**Authors:** Raíssa Bernardes da Silva, Willian dos Reis Bertoldo, Lucila Langoni Naves, Fernanda Bernadelli de Vito, Jeziel Dener Damasceno, Luiz Ricardo Orsini Tosi, Carlos Renato Machado, André Luiz Pedrosa

**Affiliations:** ^1^ Departamento de Bioquímica, Farmacologia e Fisiologia, Instituto de Ciências Biológicas e Naturais, Universidade Federal do Triângulo Mineiro, Uberaba, Brazil; ^2^ Departamento de Bioquímica e Imunologia, Instituto de Ciências Biológicas, Universidade Federal de Minas Gerais, Belo Horizonte, Brazil; ^3^ Departamento de Clínica Médica, Instituto de Ciências da Saúde, Universidade Federal do Triângulo Mineiro, Uberaba, Brazil; ^4^ Institute of Infection, Immunity and Inflammation, University of Glasgow, Glasgow, United Kingdom; ^5^ Departamento de Biologia Celular e Molecular e Bioagentes Patogênicos, Faculdade de Medicina de Ribeirão Preto, Universidade de São Paulo, Ribeirão Preto, Brazil

**Keywords:** *Leishmania major (L. major)*, DNA repair, exonuclease 1, ataxia telangiectasia mutated (ATM), ataxia telangiectasia and Rad3 related kinase (ATR)

## Abstract

*Leishmania* parasites are the causative agents of a group of neglected tropical diseases known as leishmaniasis. The molecular mechanisms employed by these parasites to adapt to the adverse conditions found in their hosts are not yet completely understood. DNA repair pathways can be used by *Leishmania* to enable survival in the interior of macrophages, where the parasite is constantly exposed to oxygen reactive species. In higher eukaryotes, DNA repair pathways are coordinated by the central protein kinases ataxia telangiectasia mutated (ATM) and ataxia telangiectasia and Rad3 related (ATR). The enzyme Exonuclease-1 (EXO1) plays important roles in DNA replication, repair, and recombination, and it can be regulated by ATM- and ATR-mediated signaling pathways. In this study, the DNA damage response pathways in promastigote forms of *L. major* were investigated using bioinformatics tools, exposure of lineages to oxidizing agents and radiation damage, treatment of cells with ATM and ATR inhibitors, and flow cytometry analysis. We demonstrated high structural and important residue conservation for the catalytic activity of the putative *Lmj*EXO1. The overexpression of putative *Lmj*EXO1 made *L. major* cells more susceptible to genotoxic damage, most likely due to the nuclease activity of this enzyme and the occurrence of hyper-resection of DNA strands. These cells could be rescued by the addition of caffeine or a selective ATM inhibitor. In contrast, ATR-specific inhibition made the control cells more susceptible to oxidative damage in an *Lmj*EXO1 overexpression-like manner. We demonstrated that ATR-specific inhibition results in the formation of extended single-stranded DNA, most likely due to EXO1 nucleasic activity. Antagonistically, ATM inhibition prevented single-strand DNA formation, which could explain the survival phenotype of lineages overexpressing *Lmj*EXO1. These results suggest that an ATM homolog in *Leishmania* could act to promote end resection by putative *Lmj*EXO1, and an ATR homologue could prevent hyper-resection, ensuring adequate repair of the parasite DNA.

## Introduction

*Leishmania* are a group of flagellated parasitic protozoans belonging to the family Trypanosomatidae (order Kinetoplastida). They are the causative agents of a collection of neglected tropical diseases, known as leishmaniases (Georgiadou et al., 2015). These parasites have a heteroxenic life cycle, alternating between different species of vertebrate and compatible Phlebotominae hosts. Evolutionarily, kinetoplastids thrived in insects as highly replicative forms owing to their nutrient-rich favorable environments. However, the nutrient accessibility of *Leishmania* spp. decreases in order to move between compartments within vertebrate and insect hosts (Alsford et al., 2012).

It is particularly challenging to understand how gene expression and cell cycle control mechanisms operate to grant the outstanding host flexibility of *Leishmania* spp. These parasites exhibit biological, metabolic, and gene expression traits, exceptionally divergent from animals, fungi, or plants (Alsford et al., 2012; Bifeld and Clos, 2015). Phenotypic changes in *Leishmania* spp., including drug resistance or tissue tropism, have been associated with their prominent genome plasticity (Zhang et al., 2014; Rastrojo et al., 2018; Patino et al., 2019). While aneuploidies are usually the cause of many cellular and developmental disorders, *Leishmania* spp. easily tolerates these genomic changes and turns them into its main tool to deal with the changing and hostile environments found within the hosts (Damasceno et al., 2018). These features raise questions regarding how these parasites maintain their genome stability and integrity in this context and how we could explore these mechanisms in alternative therapeutic strategies for leishmaniasis.

DNA repair nucleases are essential enzymes for living cells and are present in every DNA processing pathway. Exonuclease-1 (EXO1) is a member of the RAD2/XPG 5’ metallonuclease superfamily and its main activity consists of catalyzing the 5’→3’ exonucleotide hydrolysis of DNA with nicks, gaps, or previously resected ends, releasing single nucleotides. Moreover, EXO1 also acts as an endonuclease for the removal of DNA 5’ flaps or pseudo-Y structures, similar to FEN1 (Shaw et al., 2017). Primary exonuclease activity is important for mismatch repair, homologous recombination, and DNA damage response pathways (Genschel et al., 2002; Nimonkar et al., 2008; Tomimatsu et al., 2012). EXO1 secondary endonuclease activity is involved in the processing of recombination intermediate structures in immunoglobulin class switching, trinucleotide repeats, DNA-RNA hybrids, including Okazaki fragments, and ribonucleotide excision repair (Vallur and Maizels, 2008; Vallur and Maizels, 2010; Levikova and Cejka, 2015).

In homologous recombination for the repair of DNA double-strand breaks (DSBs), EXO1 excises mononucleotides in the 5’→3’ direction of a 3’ single-strand stretch previously processed by the Mre11-Rad50-Xrs2-Sae2 complex. Nucleotide excision provides a 3’ ssDNA stretch long enough for the association of repair proteins, including RPA and the recombinase Rad51 (Symington, 2016). EXO1 also plays a role in DNA damage response. The end resection performed by EXO1 stimulates a transition from ATM-dependent to robust ATR-dependent checkpoint signaling, two central protein kinases coordinating DNA damage response to DSBs, and DNA replication stress (El-Shemerly et al., 2008; Tomimatsu et al., 2017). ATM and ATR signaling provide the time and means for the cell to recover from a DNA injury by inducing cell cycle arrest and DNA repair by the appropriate molecular mechanisms. If the damage exceeds the cell repair capacity, or the cell cycle arrest is canceled prematurely, cells can accumulate DNA damage, which in turn can result in mitotic problems or even cell death (Benada and Macurek, 2015).

In a previous study, we used specific inhibitors of human ATR (ATRi) and ATM (ATMi) to evaluate the effects of oxidizing agents in *L. major* (Da Silva et al., 2018). *L. major* promastigote forms pre-treated independently with ATRi and ATMi showed a higher sensitivity to hydrogen peroxide (H_2_O_2_), demonstrating that these inhibitors could disrupt key metabolic pathways associated with the putative *Lmj*ATR and *Lmj*ATM homologues. These observations lead us to hypothesize whether a putative *L. major* Exo1 is involved in the repair of damage caused by H_2_O_2_ in the parasite. In this study, we offer novel insights into this process, using a *L. major* lineage overexpressing the putative EXO1 protozoan homologue, which exhibited marked hypersensitivity to damage induced by ionizing radiation and H_2_O_2_, when compared to the control lineages. The findings of this work could help shed light on the complex metabolic pathways responsible for the genome maintenance of *Leishmania* and its prominent capacity to adapt to the adverse conditions found throughout its life cycle.

## Materials and Methods

### Nucleic Acids Purification and Manipulation

Genomic DNA of 10^8^ log-phase *L. major* promastigote forms was extracted using the alkaline lysis method (Kapler et al., 1990). The *Lmj*EXO1 coding sequence was PCR-amplified using 25 pmol of each primer (**Supplementary Table 1**), 1.0 unit Pfu DNA polymerase and following manufacturer’s instructions (Promega, Wisconsin, USA). Thermocycling was performed using a PTC-200 thermocycler (Bio-Rad, California), with the following conditions: initial denaturation at 95°C for 5 min; 30 cycles of denaturation at 95°C for 30 s, annealing at 50.2°C for 30 s, extension at 68°C for 3 min, and a final extension at 68°C for 10 min. The resulting 3,042 bp PCR products were visualized in 0.8% (p/v) agarose gel (**Supplementary Figure 1A**).

The PCR product corresponding to the LmjEXO1 coding sequence was ligated into pGEM-T-Easy to generate pGEM-LmjEXO1. Ligation products were transformed into electrocompetent *E. coli* DH10B bacterial cells by electroporation at 2.5 V/cm in Gene Pulser (BioRad). Bacterial clones were selected on LB plates containing 100 μg/mL ampicillin.

pGEM-LmjEXO1 was digested using NotI and SspI endonucleases (**Supplementary Figures 1B, D**). The insert ends were filled using Klenow Fragment (Fermentas, Lithuania) and ligated into the SmaI-digested *Leishmania* expression vector pXG1NEO, generating pXG1NEO-LmjEXO1 (**Supplementary Figures 1C, D**).

Both pGEM-LmjEXO1 and pXG1NEO-LmjEXO1 were sequenced using a primer walking strategy (**Supplementary Table 1**). The nucleotide sequences were analyzed using an ABI PRISM 3130xl Genetic Analyzer Sequencer (Applied Biosystems).

### Transfection in *L. major* Promastigote Forms

The plasmids pXG1NEO and pXG1NEO-LmjEXO1 were transfected into *L. major* CC1 cells by electroporation (Kapler et al., 1990). Parasite colonies were transferred to 1.0 mL M199 media containing 25 μg/mL G418, and after 7 days of culture growth in liquid media, cells were transferred to 25 cm^2^ tissue flasks containing 10 mL of M199 media containing 25 μg/mL G418.

### Total RNA Extraction and cDNA Synthesis

Total RNA was extracted from 3 × 10^7^ *L. major* promastigote forms using the SV Total RNA Isolation System kit (Promega, Madison, WI, USA), following the manufacturer’s protocols. RNA samples were stored at −80°C until use.

The purity and quantification of RNA samples were obtained by spectrophotometry, using NanoDrop (Thermo Scientific, Massachusetts, USA). Complementary DNAs (cDNA) to the total RNA extracted previously were synthesized using kit GoScript Reverse Transcription System (Promega, Wisconsin, USA). One hundred nanograms of total RNA were incubated with 0.5 μg random primers and reverse transcriptase reaction was performed following the kit manufacturer´s protocol.

### Putative *LmjEXO1* Gene and Transcript Quantification by qPCR

The primer sequences used for quantitative PCR (qPCR), including their amplification efficiencies, are listed in **Supplementary Table 1**. The relative mean copy number of the putative *LmjEXO1* gene per promastigote form was determined using the Maxima SYBR Green qPCR Master Mix (Thermo Scientific, Massachusetts, USA). The relative quantification of putative *LmjEXO1* transcript levels was performed using the GoTaq qPCR Master Mix kit (Promega, Wisconsin, USA). For both quantification reactions, the amplification conditions were as follows: initial denaturation at 94°C for 10 min; 40 cycles of denaturation at 94°C for 15 s, primer association and extension at 60°C for 60 s; dissociation curve for two cycles at 94°C for 15 s and at 60°C for 15 s. Two independent qPCR experiments were performed for the putative *LmjEXO1* gene and transcript quantification. The reactions were set as technical triplicates for each experiment. All reactions were performed using a thermocycler 7500 Fast Real-Time System (Applied Biosystems, California, USA).

Relative quantification was performed as previously described (Livak and Schmittgen, 2001) (**Supplementary Figure 2**). For the relative mean copy number of the putative *LmjEXO1* gene per promastigote form, the 2^-ΔΔCt^ method was used. For the relative quantification of the putative *LmjEXO1* transcript level for each *L. major* lineage, the threshold cycles of putative *LmjEXO1* cDNA were normalized to those obtained for glucose-6-phosphate dehydrogenase (G6PD), which was used as an endogenous control.

### Prediction of the Three-Dimensional Model of *Lmj*EXO1 by Computational Modelling

We used the human Exonuclease-1 protein sequence (*Hs*EXO1; accession number NP_003677) as a query in a position-specific iterated BLAST search (PSI-BLAST) in order to rescue a conserved hypothetical *L. major* protein (TritrypDB code *Lmj*F.23.1270), which had the best hit with *HsEXO1* (E-value 1 × 10^-21^). We considered this sequence a putative for *L. major* Exonuclease-1 (*LmjEXO1*) ortholog; therefore, it was used for the subsequent analysis.

DISOPRED3 (http://bioinf.cs.ucl.ac.uk/psipred/) (Jones and Cozzetto, 2015) was used to set a boundary for the conserved N-terminal region of the predicted protein LmjExo1. A threshold of 0.5 was set as the minimum probability for a region to be predicted to be disordered. We considered the first 535 residues to be conserved and possibly prone to computational modeling (**Supplementary Figure 3A**). The model for the predicted tertiary structure of the *Lmj*EXO1 N-terminal region was built using a combined strategy of comparative and *ab initio* modeling from the ROBETTA server (http://robetta.bakerlab.org/) (Raman et al., 2009; Song et al., 2013). The model was built using an alignment with a set of sequences from experimentally defined structures, available at the Protein Data Bank (PDB) (https://www.rcsb.org/) (**Supplementary Figure 3**): human EXO1 [(PDB accession codes 3QEA and 3QE9 (Orans et al., 2011); 5V04, 5V09, 5V0C, 5UZV (Shi et al., 2017)], *Archaeoglobus fulgidus* FEN-1 [1RXW (Chapados et al., 2004)]; *Homo sapiens* FEN-1 [3Q8M (Tsutakawa et al., 2011)], and *Escherichia coli* FEN-1 [5KSE, 5K97, 5UM9 (Tsutakawa et al., 2017)].

Five models were built and analyzed using the MolProbity tool for the election, refinement, and validation of the best model (Chen et al., 2010). The parameters used for the analysis were as follows: (a) Ramachandran validation of the protein model backbone; (b) clash score; (c) rotamers; (d) carbon beta deviation; and (e) cis-peptide analysis (**Supplementary Figures 3B, C**) (Word et al., 1999; Lovell et al., 2003; Chen et al., 2010). The selected structure for *the L. major* N-terminal region was aligned with the experimentally defined structure of *Hs*EXO1 [PDB 5V06 (Shi et al., 2017)], using the Protein Structure Comparison Tool v.4.2.0 and combinatory extension algorithm (jCE) (Shindyalov and Bourne, 1998; Prlić et al., 2010).

### Molecular Docking of Macromolecules and Metallic Ions

A DNA molecule with an extended 3’ end (sequence 5’→3’ CGCTAGTCGACAT; complementary chain sequence 5’→3’ TCGACTAGCG), the prototypical substrate for EXO1, and the metallic ions manganese (Mn^2+^) and sodium (Na^+^) were inserted into the *Lmj*EXO1 model using the MatchMaker tool of Chimera v. 1.11.2 (http://www.rbvi.ucsf.edu/chimera/). The Needleman-Wunsch global alignment algorithm and substitution matrix BLOSUM-62 were used to build the structural alignment and superposition of the protozoan protein model with *Hs*EXO1 (Pettersen et al., 2004; Meng et al., 2006). A threshold of 2.0 Å was used to limit structural alignment.

### Cell Culture Assays

*Escherichia coli* DH10B was maintained at 37°C in LB media [0.5% (p/v) yeast extract; 1.0% (p/v) tryptone; and 1.0% (p/v) NaCl], with the addition or not of 1.5% (p/v) bacteriological agar.

Wild-type (WT) promastigote forms of *L. major* clone CC1 were maintained in M199 medium supplemented as described (Kapler et al., 1990). Parasite cells were cultured at 28°C in a BOD incubator, with media replacement every seven days.

*L. major* lineages transfected with the expression vector *pXG1-NEO*, named “*L. major* CC1 [*pXG1-NEO*]” (control), and with the expression vector *pXG1NEO LmjEXO1* (**Supplementary Figure 1**), named “*L. major* CC1 [*pXG1 NEO LmjEXO1*],” were maintained in the culture media described previously for *L. major* CC1 *wild-type*, with the addition of 25 μg/mL geneticin (G418). Stock solutions of ATRi (VE-821; Sigma-Aldrich) and ATMi (KU-55933; Sigma-Aldrich) were diluted in DMSO. Caffeine was diluted in the standard M199 medium. Stock solutions were maintained at ‒20°C and protected from light to prevent any degradation of the drugs. Inhibitors were added to the supplemented M199 when indicated.

### Growth and Susceptibility of *L. major* Lineages to Ionizing Radiation

To evaluate the growth and the susceptibility of *L. major* lineages after exposure to ionizing radiation (IR), promastigote forms from the lineages WT, pXG1, and EXO1 in log-phase were collected and resuspended to achieve the concentration of 1‒3 × 10^6^ parasites/mL in a volume of 30 mL of media in 75 cm^2^ tissue flasks. The parasites were resuspended in standard culture media for the control groups or media supplemented with 5 mM caffeine for the treated groups. The cultures were incubated for 1 h at 28°C and then exposed to a 500 Gy dose of gamma radiation from a cobalt-60 (^60^Co) source, with a transfer rate of 3.112,56 Gy/h (total exposure time: 9’40”). Irradiation was performed at the Gamma Irradiation Laboratory at the Nuclear Technology Development Center (LIG/CDTN) at the Universidade Federal de Minas Gerais, MG, Brazil (Garcia et al., 2016).

The cultures were incubated at 28°C for 5 h, and the parasites were counted using a hemocytometer. Then, they were washed with media M199 at 2,000 × *g* for 10 min at 4°C for the complete removal of the drugs. The parasites were resuspended in the appropriate culture medium for each lineage, as previously described. Subsequently, the cultures were aliquoted in 24-well plates in triplicate, and the plates were incubated at 28°C. The relative growth and susceptibility curves were determined with daily counts during the same day period, using a hemocytometer, until the IR-exposed promastigotes reached a stationary phase, defined as two days of similar counts.

Data are expressed as the mean of the death percentage of the initial parasite population or percentage of the maximum growth ± standard error of the means obtained from two independent experiments. One hundred percent growth was considered for the maximum parasite concentration achieved by cultures non-treated with genotoxic compounds or non-exposed to IR. Conversely, 100% death was considered to be the total loss of parasite cells from the starting population. These normalizations were done to equalize the initial parasite populations of the groups WT, pXG1, and EXO1, which could vary owing to growth rate variations of these lineages.

### Growth of *L. major* Lineages After Exposure to H_2_O_2_

To evaluate the effect of ATR and ATM inhibitors in the growth of *L. major* lineages exposed to hydrogen peroxide, promastigote forms of WT, pXG1, and EXO1 lineages in log-phase were collected and centrifuged at 2,000 × *g* for 10 minutes and at 4°C for the complete removal of the culture media. The parasites were then resuspended to a concentration of 5 × 10^6^ cells/mL in media containing 10 μM ATRi, 10 μM ATMi, a combination of these two (10 μM ATRi plus 10 μM ATMi), or 5 mM caffeine. The parasites were incubated at 28°C for 1 h before the addition of 500 μM H_2_O_2_. The cells were incubated under these conditions for 20 min without agitation, and protected from light to prevent the decomposition of H_2_O_2_. The cultures were then centrifuged at 2,000 × *g* for 10 min at 4°C. The supernatant was completely washed out and replaced with the same volume of appropriate culture media for each lineage. Samples were aliquoted in 24-well plates in triplicate and incubated at 28°C, protected from light, for 72 h. After this period, parasites were counted using a hemocytometer.

One hundred percent growth was considered for the maximum parasite concentration achieved by cultures not treated and not exposed to H_2_O_2_. The parasite population of the treated cultures was relative to that of the population. Because of possible variations in the growth rates of the lineages WT, pXG1, and EXO1 the 100% growth percentage was considered for each lineage individually. Data are presented as the mean ± standard error (SE) of the relative growth percentages obtained from three independent experiments.

### Biostatistics Analysis

All statistical tests were performed using IBM SPSS v. 20.0.0 and the graphs were constructed using GraphPad Prism v. 5.03. All test results were considered significant when *P* < 0.05. For the statistical tests of means, Levene’s homogeneity test and Shapiro-Wilk’s normality test were used for the pre-assessment of the study variables. Both tests were considered statistically non-significant; therefore, the following parametric tests were used. Two-way ANOVA was used for the comparative analysis of the relative growth of the three *L. major* lineages (i.e. WT, pXG1, and EXO1) when treated with inhibitors and/or exposed to hydrogen peroxide. The Bonferroni *post-hoc* test was used to compare lineages within treatment groups, while Dunnett’s *post-hoc* test was used to compare treatment groups. In this case, the non-treated (“NT”) group was considered the control group. Flow cytometry data, that is, the percentage of cells in each cell cycle stage (sub-G1; G1, S, G2, and >G2) were analyzed using the Chi-square test.

## Results

### Computational Modelling of a Three-Dimensional Structure for the Putative *Lmj*EXO1

The putative Exonuclease-1 homologue in *L. major* (*Lmj*EXO1) could be divided into two main components: (a) an N-terminal region, predicted to contain a conserved catalytic domain; (b) a C-terminal region, characterized by a putative intrinsically disordered segment (**Supplementary Figure 3A**). With a predicted total of 1,013 amino acids, the first 535 residues of this predicted enzyme were used for computational modeling. The three-dimensional model for putative *Lmj*EXO1 was built from the alignment with 26 experimentally solved structures deposited in PDB, 12 of which were unique structures (**Supplementary Figure 4**). In addition to the low sequence similarity, long insertion segments were predicted to be present in *Lmj*EXO1. The predicted inserts ranged from amino acid residues 46–95, 154–183, 272–363, and 500–535. These residues could not be resolved by comparative modeling; therefore, they were modeled by *ab initio* strategy, resulting in the prediction of additional secondary structures.

**Figure 1:**
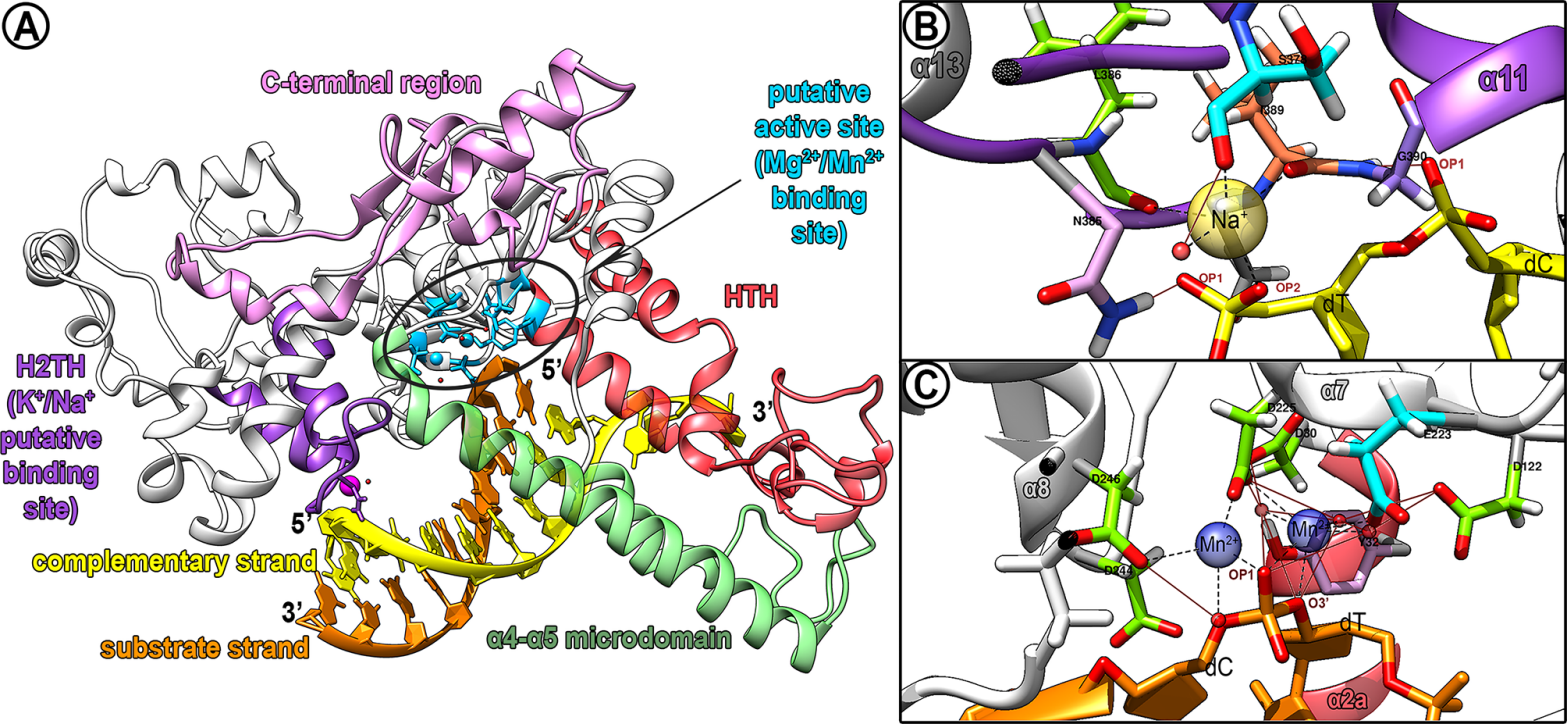
Putative catalytic domain of LmjEXO1 and predicted interactions with substrate DNA and metallic ions. **(A)** Prediction of the main regions of the catalytic core of LmjEXO1 by structural alignment with HsEXO1 (SHI et al, 2017), i.e.: active site (sky blue), possibly containing sites for bivalent ligands including Mg^2+^ and/or Mn^2+^ (blue spheres); domain helix-two-turn-helix (H2TH, purple); with a putative monovalent ligand including sodium or potassium (magenta sphere); helix-turn-helix wedge (HTH, red); α4-α5 microdomain (green); C-terminal region (pink). The small red spheres constitute putative water molecules. **(B, C)** The inside of the predicted domain H2TH and the putative active site of LmjEXO1 were zoomed in. The side chains of the residues predicted in the coordination of the metallic ions are shown by sticks. Predicted hydrogen bonds are presented as brown lines, whereas putative pseudobonds for metallic ions coordination are displayed as dashed lines in black. Water molecules possibly involved in the interaction are shown as red translucent spheres. Oxygen atoms are shown as red sticks; nitrogen atoms are displayed as blue sticks, and hydrogen atoms are shown in white. Alpha-helixes predicted around the ligand sites are also displayed. **(B)** The predicted site for monovalent K+/Na+ ligands (yellow translucent sphere) is displayed. Predicted residues in the coordination: S378 (cyan); N385 (pink); L386 (green); I389 (orange); G390 (purple). Yellow sticks represent the phosphodiester backbone of the complementary strand (non substrate) of the DNA. OP1 and OP2: oxygen atoms of phosphate groups. **(C)** The predicted active site is displayed with two bivalent cations (Mg^2+^ and/or Mn^2+^), presented as blue translucent spheres. Residues predicted in the coordination: D30, D122, D225, D244 and D246 (green); E223 (cyan); Y32 (purple). Orange sticks represent the phosphodiester backbone of the substrate DNA strand.

The structure predicted for putative *Lmj*EXO1 revealed conservation of its global aspect, a bean-shaped structure resembling its human homologue (**Figure 1A**). Ramachandran’s validation of this model qualified 96.1% (512/533) of the analyzed residues in favorable regions, and 99.8% (532/533) of the residues in the allowed regions (**Supplementary Figure 3B**). The only predicted outlier residue was an isoleucine, the third residue in *the Lmj*EXO1 sequence, which does not seem to participate in any of the predicted main structures of this protein. All residues of the model presented a predicted carbon beta deviation smaller than 0.25 Å, being classified in a favorable position, 19 of them with a deviation >0.125 Å (**Supplementary Figure 3C**). The overall analysis of these parameters placed the proposed three-dimensional model of putative *Lmj*EXO1 in the 100^th^ percentile from a set of 27,675 structures deposited in the MolProbity databank.

### Predicted Structural Domains of Putative *Lmj*EXO1

The structural alignment of the model proposed for putative *Lmj*EXO1 with the experimentally resolved structure of human EXO1 [*Hs*EXO1; PDB accession code 5V06 (Shi et al., 2017)] was built using Protein Structure Comparison Tool v.4.2.0. The three-dimensional structures of the two proteins presented a root mean square deviation (RMSD) of 1.72 (**Supplementary Figure 5**). The comparison of the aligned structures and their sequences allowed the prediction of important domains in the putative *Lmj*EXO1 N-terminal region (**Figure 1A**): (i) the domain helix-two-turns-helix (H2TH), ranging from residue Y368 to H400 (α10 and α11 structures), which, in the human homologue, binds to the DNA duplex and contains a coordination site for monovalent cations Na^+^ or K^+^; (ii) the active site, found in double strand/single strand DNA junction, where the 5’ end processing is predicted to occur; (iii) two long helixes linked by a loop to form the α4-α5 microdomain, ranging from residues P127 to K193; (iv) two helixes linked by loops, in the shape of a wedge, constituting the helix-turn-helix (HTH) domain and ranging from residue M31 to F113. The majority of the residues contained in this region of the putative *Lmj*EXO1 are hydrophobic, which are responsible for the 3’ ssDNA extension bending from the junction point in the human homologue enzyme.

Most differences between the predicted model of *Lmj*EXO1 and *Hs*EXO1 arise from the insertions found in the protozoan homologue. These segments, solved by *ab initio* strategy, were predicted to form long loops with poorly defined alpha-helices (**Figure 1A** and **Supplementary Figure 5**). The average estimated error for the position of the residues contained in these regions is 14.65 Å, and these regions are predicted to be intrinsically disordered (**Supplementary Figure 3A**).

Important residues for the catalytic activity of the human enzyme *Hs*EXO1 were conserved in the putative *Lmj*EXO1 (**Supplementary Figure 6**). For instance, the amino acid residues D30, D152, D171, D173, and D225 of the human homologue are responsible for the coordination of the bivalent cations of Mg^2+^ or Mn^2+^ (Orans et al., 2011), and they are structurally conserved in the parasite enzyme residues D30, D225, D244, D246, and D381). The carbonyl groups of *Hs*EXO1 residues S222 and I233 and the hydroxyl group of S229 are responsible for the interaction with the monovalent cations K^+^ or Na^+^. The first two residues are predicted to be conserved in putative *Lmj*EXO1 (S378 and I389), whereas the last residue appears to be replaced by an asparagine (N385). However, the hydroxyl group of N385 could coordinate with the monovalent cation K^+^/Na^+^, similar to the serine in the human enzyme. Residues H36, K85, R92, R95, R96, and K185 of *Hs*EXO1 interact with the complementary, non-substrate DNA strand and have corresponding residues in the putative *Lmj*EXO1 (H36, K129, R136, R139, R140, and K258). Conversely, three out of the eight *Hs*EXO1 residues that interact directly with the substrate DNA strand (K37, K61, R121, R231, G232, G234, A236, and K237) are conserved in the parasite enzyme (R387, G390, and K393). The five substituted residues were predicted to be either conservative substitutions (*Lmj*EXO1 R37 and *Hs*EXO1 K37; *Lmj*EXO1 K193 and *Hs*EXO1 R121; *Lmj*EXO1 S388 and *Hs*EXO1 G232) or non-conservative replacements (*Lmj*EXO1 D105 and *Hs*EXO1 K61; *Lmj*EXO1 K392 and *Hs*EXO1 A236). Lastly, the G79 residue, located at the end of the *Hs*EXO1 β3 sheet, acts as a hinge and is responsible for the coordinated movement of the mobile arc (α4-α5 microdomain) of the human enzyme. This residue is predicted to be conserved in putative *Lmj*EXO1 (G123), and it is also found at the end of a β-sheet, just before the α4-α5 microdomain of the parasite enzyme.

### Predicted Active Site of Putative *Lmj*EXO1 and Interaction With Metallic Ions and Substrate DNA

EXO1 is a metalloprotease with two regions associated with metallic ions (Orans et al., 2011; Shi et al., 2017). The first one is constituted by the H2TH motif, while the second is found inside the active site of the enzyme. In *L. major*, the predicted H2TH domain may contain a monovalent cation (Na^+^ in the proposed model) (**Figure 1B**). Moreover, this domain is predicted to form hydrogen bonds between the phosphate oxygens (OP1 and OP2 in the model) of cytosine or thymine and the amino groups of the main chain of residue G390 and side chain of residue N385. It was also predicted that the cation Na^+^ is coordinated by the carbonyl groups of the residues S378, L386, and I389, all of which are contained in the loops of the H2TH motif. Phosphate oxygen (OP2 in the model) and a water molecule bound to the complex by hydrogen bound to the carbonyl group of residue S378, could also participate in the coordination of the monovalent metallic ion.

The predicted active site of putative *Lmj*EXO1 can be found in the double strand/single-strand DNA junction, in a similar fashion to its human counterpart (**Figure 1C**). It was predicted to accommodate two bivalent cations, Mg^2+^ and/or Mn^2+^, in the proposed model of *Lmj*EXO1. These metal ions can be coordinated by the side chains of residues E223, D225, and D246, or by water molecules bound by hydrogen bonds to residues D30, Y32, D122, E223, D225, and D246. Mn2^+^ also appears to be coordinated by nucleotides in the substrate DNA strand, whether directly by the phosphate oxygen (OP1 in the model), or indirectly by water molecules bound by hydrogen to the 3’ oxygen (O3’).

### Overexpression of *Lmj*EXO1 in *L. major* Promastigotes Increases Parasite Sensitivity to Ionizing Radiation

**Figure 2:**
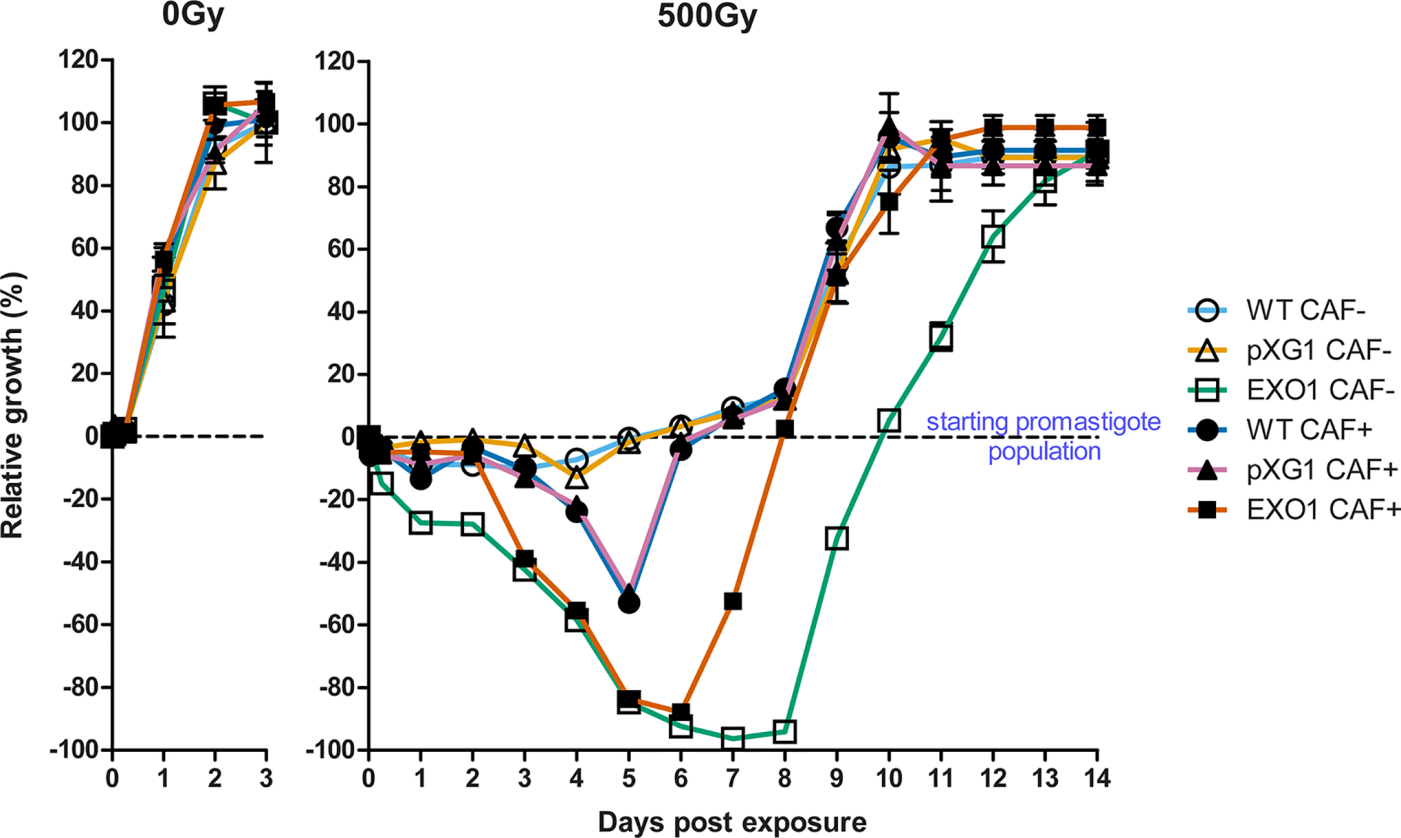
Growth and susceptibility curves of *L. major* lineages exposed to ionizing radiation. The graphs display how the *L. major* cell population of the three lineages evolved after the exposure to ionizing radiation (IR) and the effects of the addition of 5 mM caffeine over that evolution. WT lineage, not treated with caffeine (WT CAF-): empty circles; episome vector control lineage, not treated with caffeine (“pXG1 CAF-”): empty triangles; LmjEXO1 overexpressor lineage, not treated with caffeine (“EXO1 CAF-”): empty squares; wild type lineage, treated with caffeine (WT CAF+): full circles; episome vector control lineage, treated with caffeine (“pXG1 CAF+”): full triangles; LmjEXO1 overexpressor lineage, treated with caffeine (“EXO1 CAF+”): full squares. The parasites were exposed to 500 Gy of gamma radiation and counted daily with hemocytometer. The data were normalized and presented as mean ± standard error for two independent experiments, each one performed in triplicate. The point 0% represented the initial parasite population, right before the irradiation (the dashed line along the graphs). Under that line (negative values), the data points represent the percentage of cell death from the initial starting point, i.e., 100% of the lower part of the graph means the complete death of initial parasite population. The superior part of the graph (positive values) represent parasite population growth, so that 100% means maximum growth, defined as the maximum quantity of parasites reached by each lineage not exposed to IR (0 Gy).

Our research group was capable of generating a *L. major* CC1 lineage overexpressing the putative *LmjEXO1* gene product from an epissomal expression vector (**Supplementary Figure 2**). In humans, exonuclease-1 acts in mismatch repair and homologous recombination, among other DNA damage repair pathways (Nielsen et al., 2004; Bolderson et al., 2010). For this reason, we decided to investigate the effect of the overexpression of putative *Lmj*EXO1 in *L. major* exposed to ionizing radiation (IR), a causative agent of DNA double-strand breaks (Borrego-Soto et al., 2015; Hur and Yoon, 2017). Promastigote forms were exposed to 500 Gy of gamma radiation and counted daily until they reached the stationary stage. A halt in the growth of wild-type (WT) and episomal vector control (“pXG1”) were observed during the five days after the genotoxic damage (**Figure 2**). The growth resumed after that period, and the parasite populations of these two lineages reached numbers similar to those of the same lineages not exposed to IR. Conversely, the parasite population of the putative *Lmj*EXO1 overexpressor lineage (“EXO1”) declined right after the irradiation, reaching an average mortality rate of 94.33% of the parasites from the initial inoculum, between the sixth and the eighth days after exposure to IR. After that period, the overexpressor lineage recovered and approximately 12 days after the exposure, it reached a population comparable to that of the control groups.

### Overexpression of Putative *Lmj*EXO1 Interfere in Cell Cycle After Exposure to Ionizing Radiation

**Figure 3:**
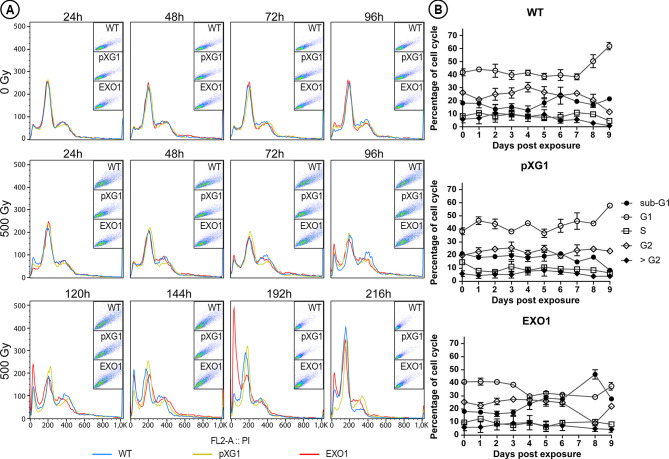
Cell cycle of the different *L. major* lineages exposed to ionizing radiation. **(A)** The histograms compare the cell populations of *L. major* wild type (WT, blue curves); episome vector control (“pXG1”, golden curves); e LmjEXO1 overexpressor lineage (“EXO1”, red curves) not exposed (“0 Gy”) and exposed to 500Gy (“500Gy”) of gamma radiation during the analyzed period. The population was distributed according to the fluorescence intensity of propidium iodide (PI) of each cell. The inset graphs at the right upper corner of each histogram represent the population of parasites from which the data was extracted for the generation of the histograms. The data are representative of two independent experiments. **(B)** The percentage of parasites in each stage of the cell cycle, extracted from the histograms, was plotted as curves to demonstrate the evolution of each *L. major* after the irradiation. Sub-G1: full circles; G1: empty circles; S: empty squares; G2: empty diamonds; > G2: full diamonds. The data was presented as mean ± standard error of the percentages obtained from the two independent experiments.

To verify whether the hypersensitivity of the putative *Lmj*EXO1 overexpressor and the growth arrest presented by the control lineages were due to changes in the progression of the cell cycle, we evaluated the effects of IR exposure on the cell cycle progression of these parasites by flow cytometry (**Figure 3A**). Parasite culture samples were collected daily after the exposure to IR, and we observed slight increases in G2 cell populations and concurrent decreases in G1 cell populations for *L. major* WT and pXG1 during four days after exposure to IR (**Figure 3B** and **Supplementary Table 2**). Thereafter, we observed a slight decrease in G2 cell populations and an increase in G1 and sub-G1 until the ninth day, when the G1 cell population increased significantly (χ^2^(8) = 33.617, *P* < 0.0005) in comparison to the cell populations of the putative *Lmj*EXO1 overexpressor lineage. No changes were observed for the other cell cycle stages (i.e., S and >G2 phases). Conversely, the *L. major* “EXO1” lineage presented a gradual increase in sub-G1 stage during the post-irradiation period and, by the eighth day of evaluation, a significant increase of the cell population at this stage was observed with a simultaneous reduction of parasites in G2 (χ^2^(8) = 35.21, *P* < 0.0005). It was not possible to retrieve data from the seventh day post-IR for the overexpressor lineage owing to the high mortality rate, so there were not enough promastigote forms for quantification by flow cytometry. By the ninth day, it was possible to observe a recovery of *L. major* “EXO1,” with significant increases in G2 cell populations, in accordance with the growth observed for this parasite lineage reported previously for this period (**Figure 2**).

### Caffeine Addition Induces Changes in Post-Irradiation Phenotype of *L. major* Lineages

In other organisms, different DNA damage response pathways are coordinated by an intricate signaling network, which maintains cellular genomic integrity through the DNA damage response (DDR), a network coordinated by the PIKK proteins ATM and ATR (Matsuoka et al., 2007; Smith et al., 2010). We decided to investigate whether the hypersensitivity phenotype presented by *L. major* “EXO1” exposed to IR could be modified by the addition of caffeine, a methylxanthine recognized as a non-selective inhibitor of these cell signaling protein kinases (Blasina et al., 1999; Sarkaria et al., 1999). Promastigote forms of the three *L. major* lineages (WT, pXG1, and EXO1) were incubated for 1 h in culture media containing 5 mM caffeine prior to the exposition to 500 Gy gamma radiation. The parasites were counted 6 h post-IR exposure and then daily until they reached the stationary phase, similar to the assay described previously.

Indeed, caffeine addition caused changes in the phenotypes of the three *L. major* lineages after exposure to IR (**Figure 2**). A gradual decline of the cell population from both WT and pXG1 control lineages treated with 5 mM caffeine was observed after irradiation, until the fifth day of evaluation, when they reassumed cell growth. The putative *Lmj*EXO1 overexpressor lineage treated with caffeine presented similar mortality rates to the same non-treated lineage — 87.8% death of initial promastigote population by the sixth day for caffeine treated “EXO1” compared to 92.4% for the non-treated “EXO1.” However, caffeine treatment before irradiation allowed the parasites from the overexpressor lineage to recover growth by the sixth day of evaluation, two days prior to the recovery observed for the non-treated counterpart.

### Specific Human ATR and ATM Inhibitors Induce Opposing Phenotypes in *L. major* Lineages

**Figure 4:**
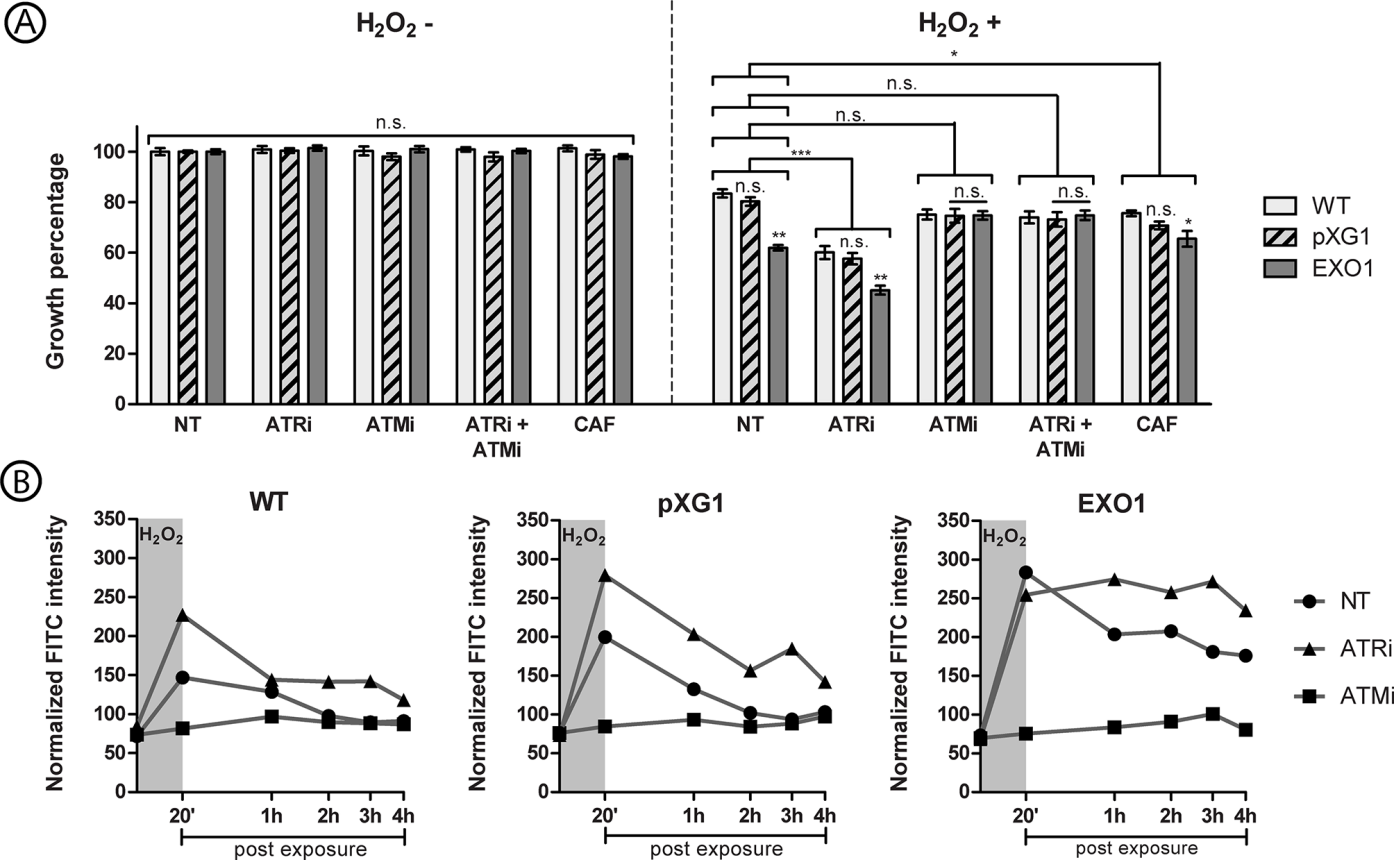
Effect of ATR and ATM inhibitors in the growth and ssDNA formation of *L. major* lineages exposed to hydrogen peroxide. **(A)** The graph display percentage of growth of *L. major* wild-type (WT, light grey columns), episome vector control (pXG1, hatched columns) and LmjEXO1 overexpressor lineage (EXO1, dark grey columns), after exposure to hydrogen peroxide (H_2_O_2_). The parasite cells were treated with 5 mM caffeine (CAF), 10 μM ATRi, 10 μM ATMi, or a combination of 10 μM ATRi and 10 μM ATMi, for 1 hour before the addition of H_2_O_2_.The parasites were then exposed to 500 μM H_2_O_2_ for 20 minutes. Cells were counted after 72 hours. The growth percentage is relative to the one of parasites not treated and not exposed to H_2_O_2_ of each lineage. The data represent mean ± standard error of three independent experiments, each one performed as triplicates. Two way analysis of variance (two-way ANOVA) is shown, and it includes the results of the *post-hoc* tests of Bonferroni (comparisons between lineages inside each treatment) and Dunnett (brackets comparing treatment groups). Control groups are the parasites not treated with inhibitors. *p < 0,05; **p < 0,005; ***p < 0,0005. **(B)** The curves exhibit the ssDNA formation kinetics in *L. major* wild type (WT), episome vector control (pXG1) e LmjEXO1 overexpressor lineage after the exposure to H_2_O_2_. Promastigote forms of *L. major* lineages were incubated during 1 hour in standard culture media (NT, circles) or media containing 10 μM ATRi (triangles) or 10 μM ATMi (squares). 500 μM H_2_O_2_ were added to the cultures, which were then incubated for 20 minutes (darker area in the graphs). The parasites were washed for complete removal of the drugs and resuspended in standard culture media. 0,5 a 2×10^7^ cells samples were collected 1 hour after the addition of inhibitors (starting point of the curves), after 20 minutes of the addition of H_2_O_2_, and then every hour after the removal of the drugs, until 4 hours post H_2_O_2_ exposure. After cell fixation, the parasites were incubated with anti-BrdU FITC conjugate for the detection of 5-IdU nucleotide. The mean fluorescence intensity of parasites with a >10^2^ UA fluorescence intensity was normalized with the mean fluorescence of parasites under that threshold. The histograms used for this quantification are presented in **Supplementary Figure 6**. The data are representative of two independent experiments and are presented as means ± standard errors of the normalized fluorescence intensities. n.s., not significant.

In a previous study by our group, we demonstrated that the treatment of *L. major* wild-type promastigotes with specific inhibitors developed for human ATR and ATM, VE-821 and KU-55933, respectively, rendered these parasites susceptible to damage induced by hydrogen peroxide (H_2_O_2_) (Da Silva et al., 2018). Therefore, we decided to investigate if these inhibitors, which now we designate “ATRi” and “ATMi,” could alter the phenotype of the *L. major* and, more specifically, of the putative *Lmj*EXO1 overexpressor lineage. *L. major* promastigote forms from WT, pXG1, and EXO1 lineages were treated with 10 μM ATRi, 10 μM ATMi, a combination of 10 μM ATRi and 10 μM ATMi, or 5 mM caffeine for one hour before the exposure to 500 μM H_2_O_2_ (**Figure 4A**).

After 72 hours of the exposure to H_2_O_2_, it was possible to observe a hypersensitivity phenotype of “EXO1” lineage to the effects induced by the oxidant agent when compared to the control lineages. Moreover, treatment with ATRi led to a significant increase in the sensitivity of all lineages to 500 μM H_2_O_2_ in comparison to non-treated lineages.

Strikingly, however, “EXO1” phenotype after exposure to IR is completely altered with the addition of ATMi, in opposition to the observed effect for ATRi treatment. This inhibitor seemed to exert a “protective” effect on the promastigotes of the overexpressor lineage exposed to H_2_O_2_, which displayed growth similar to that of the control lineages. Moreover, the combination of the two inhibitors did not seem to interfere with the phenotype observed for parasites treated only with ATMi. Finally, caffeine treatment allowed a growth rate for the “EXO1” lineage exposed to H_2_O_2_ in a similar fashion to the “pXG1” control.

### Specific Human ATR and ATM Inhibitors Render Different Effects Over ssDNA Formation in *L. major* Lineages

The opposing effects of ATRi and ATMi on the phenotype of the putative *Lmj*EXO1 overexpressor lineage suggest that these two protein kinases modulate the parasite enzyme functions in different ways. DNA end processing to generate extended single-strand filaments can be considered the main function of EXO1, as seen in other organisms (Shaw et al., 2017). Therefore, we used an ssDNA detection assay by flow cytometry to investigate whether the phenotypes displayed by the *L. major* “EXO1” lineage could be associated with different patterns of ssDNA generation. In this assay, 5-iodo-2’-deoxyuridine (5-IdU) incorporated into the genome can be detected by a FITC conjugate if this nucleotide is “available for binding,” that is, in exposed ssDNA filaments. *L. major* promastigotes were treated with 10 μM ATRi or 10 μM ATMi for one hour before the addition of 500 μM H_2_O_2_. Samples were collected 1 h after the addition of inhibitors, 20 min after the addition of H_2_O_2_, and every hour after the removal of drugs, in order to build kinetic curves of ssDNA formation (**Figure 4B**). When exposed to H_2_O_2_, we observed a parasite population peak, with FITC fluorescence intensity (FI) higher than 10^2^ UA (**Supplementary Figures 7A-C**). We used this value as the threshold for the analysis in order to discard any ssDNA formation due to normal cell events, including transcription or replication. Moreover, the mean FI for the parasite population above 10² UA was normalized to the mean FI of the population under this threshold, aiming to disregard any excess of fluorescence markers.

After 20 min of exposure to H_2_O_2_, large ssDNA formation was observed in promastigotes of the putative *Lmj*EXO1 overexpressor lineage (**Figure 4B**). On average, non-treated parasites from the “EXO1” lineage presented a normalized FI of 1.42 times the observed for lineages “pXG1” and almost double the normalized FI of wild-type parasites. After that period, a gradual decrease in the amount of ssDNA was observed for “EXO1” parasites; however, this was not enough to achieve ssDNA levels before the addition of H_2_O_2_. Conversely, an effective reduction of ssDNA was observed in the control lineages, which returned to the pre-exposure values after approximately 2 h.

The noteworthy effects were, in fact, associated with the addition of ATRi and ATMi, which substantially altered this scenario (**Figure 4B**). After 20 min of exposure to H_2_O_2_, WT and pXG1 (controls) treated with ATRi showed higher ssDNA formation rates than their non-treated counterparts. Moreover, the ssDNA formation in WT and pXG1 treated with ATRi is similar to what was observed for the putative *Lmj*EXO1 overexpressor lineage. This, in turn, does not seem to show observable changes in ssDNA formation between ATRi-treated and non-treated groups. Additionally, in all three lineages, an apparent persistence of ssDNA seemed to occur after the 4-hour period, when the ssDNA levels had not yet reached the pre-exposure values. This persistence seemed to be more intense for the “EXO1” lineage, since no observable reductions in ssDNA levels occurred during the analyzed period.

Contrary to the effect observed in promastigotes treated with ATRi, pre-treatment with ATMi seemed to prevent additional ssDNA formation after exposure to the oxidant agent. After 20 min of exposure to H_2_O_2_, no peaks of ssDNA formation resembled the previous cases (**Figure 4B** and **Supplementary Figures 7A–C**). In all three *L. major* lineages, only a slight increase in ssDNA levels was observed during the four-hour period; it did not surpass 1.4 times the pre-exposure levels for any of the parasite lineages. These findings suggest that the putative *Lmj*ATM and *Lmj*ATR could influence the DNA damage repair process after exposure to an oxidant agent, either favoring (in case of *Lmj*ATM) or preventing (in case of *Lmj*ATR) the extended ssDNA formation, possibly derived from the activity of putative *Lmj*EXO1. These observations could help explain the reasons underlying the hypersensitivity of the putative *Lmj*EXO1 overexpressor lineage after exposure to different genotoxic damages.

## Discussion

The RAD2/XPG family is an ancestral protein family conserved throughout evolution, with members encountered in many species, from phages to humans (Lieber, 1997; Ceska and Sayers, 1998; Lee and Wilson, 1999). These members can be classified into four classes: XPG (Class I), FEN-1 (Class II), EXO-1 (Class III), and Yen1/GEN1 (Class IV) (Ishikawa et al., 2004). These enzymes act on the three fundamental axes of genome maintenance: DNA replication, repair, and recombination (Qiu et al., 1999; Genschel et al., 2002; Nimonkar et al., 2008; Potenski et al., 2014). Their common catalytic core is contained in the N-terminal region, and it processes a large variety of DNA substrates, including nicks, gaps, 5’-flaps, Holliday junctions, and bubbles (Evans et al., 1997; Lee and Wilson, 1999; Ip et al., 2008; Keijzers and Vilhelm, 2015). In the present work, we proposed a tertiary structure model for the catalytic core of the putative Exonuclease-1 homologue in *L. major* (*Lmj*EXO1), based on the structural conservation of the N-terminal region. Two major features of the parasite putative enzyme turned clear and were further evidenced by the predicted three-dimensional structure. The first one suggests that the main domains responsible for the catalytic activity of human EXO1 (Orans et al., 2011), that is, the helicoidal arc (α4-α5 microdomain), the HTH hydrophobic wedge (α2-α3 helix-turn-helix), and H2TH motif (helix-two-turns-helix), are structurally conserved in the parasite enzyme.

The increase in the size of the predicted helicoidal and hydrophobic domains is directly associated with the second feature found in the sequence alignment: the long insertion segments that are present in the parasite putative enzyme. The segment formed by residues R46 to I95 is contained in the predicted HTH domain, while the insertion L154 until P183 constitutes the helicoidal domain. These insertions may be associated with the functions of these domains. For instance, the helices α2 and α3 in human exonuclease are located in the boundaries of DNA pre- and post-cleavage regions; their hydrophobic interactions with the DNA cause a sharp fold in the 3’ DNA strand extended by resection and prevent binding to unprocessed dsDNA (Chapados et al., 2004; Orans et al., 2011). It is also important to consider that these insertions are represented by stretches predicted to be structurally disordered. Although not seen in *Hs*EXO1, this feature does not seem to be exclusive to the putative *Lmj*EXO1. In 5’ nucleases, the mobility and degree of disorder of the α4 and α5 helices, which constitute the mobile helicoidal arc, for instance, may vary significantly. These regions are disordered in many homologous structures, including the bacteriophage T4 5’ nuclease and FEN1 from archaea and humans (Sakurai et al., 2005; Doré et al., 2006; Devos et al., 2007). It has been suggested that transitions of these regions from structurally disordered to ordered could occur during binding to flap substrates, as seen in FEN-1, and they could help in the flexibility of these enzymes to different substrates (Sayers and Artymiuk, 1998).

We demonstrated that the overexpression of the putative *Lmj*EXO1 enzyme renders the parasite significantly more susceptible to genotoxic damage induced by ionizing radiation. In other organisms, EXO1 is fundamental for DNA repair, and its deficiency is associated with increased susceptibility to lymphomas in knockout mice (Wei et al., 2003; Schaetzlein et al., 2013). In contrast, EXO1 is generally expressed at low levels, independent of the proliferative state or the cell cycle, and increases in its expression are harmful to the cell (Fernandez-Capetillo and Nussenzweig, 2013; Keijzers et al., 2018). After activation, this enzyme is tightly controlled to prevent excessive end resection. It has been proposed that long extensions of 3’ ssDNA are a threat to the cell because they are susceptible to breakage and could lead to global genomic instability by the depletion of the existing RPA molecules, which are recruited to the nascent ssDNA strand (Toledo et al., 2013). Moreover, DNA hyper-resection could also lead to a transition in the DSB repair pathway from error-free homologous recombination (HR) to the highly deleterious single-strand annealing pathway (SSA) (Zong et al., 2016). SSA is a process associated with repetitive homolog sequences around a DSB (Bhargava et al., 2016). The result of DSB repair by SSA is a rearrangement between homolog repetitions, with genetic information losses. For this reason, we could not discard the hypothesis that DNA repair by SSA may be induced by the overexpression of putative *Lmj*EXO1 in the parasite, which could help explain the hypersensitivity of this lineage to the genotoxic damage caused by IR.

We have further observed that the control lineages WT and pXG1, treated with 5 mM caffeine prior to the exposure to 500 Gy γ-radiation, decreased in population until the fifth day post-IR, with a subsequent growth recovery. Caffeine is a non-selective inhibitor of the cell signaling protein kinases, ATR and ATM. It inhibits cell proliferation, prevents delays in cell cycle progression, and increases the cytotoxic effect of radiation and chemotherapeutic compounds (Lau and Pardee, 1982; Levi-Schaffer and Touitou, 1991). However, the most noticeable effect was the faster recovery of the putative *Lmj*EXO1 overexpressor lineage because of caffeine treatment. We first hypothesized that hypersensitivity of the overexpressor lineage could be associated with cell signaling promoted by an ATR homologue in *L. major*. In other organisms, the activation of the ATR-ATRIP complex is mediated by the enzyme activity, which generates 3’ ssDNA tails to be recovered by RPA; ATR phosphorylates EXO1, leading to its ubiquitination and degradation. It is believed that this mechanism serves as a negative feedback of ATR, in order to regulate its own activation, to limit the DNA damage checkpoint signaling, and to prevent excessive end resection that could generate products unsuitable for repair by HR (Rossiello et al., 2014; Chen et al., 2015; Tomimatsu et al., 2017). We believe that this theory could help explain the hypersensitivity phenotype of the *L. major* “EXO1” lineage after damage induced by ionizing radiation, but not the faster recovery of this lineage promoted by the addition of caffeine. In fact, caffeine can prevent cell cycle arrest promoted by excessive ATR signaling. However, a deficiency in the activity of this regulatory protein kinase could exacerbate the hyper-resection of DNA strands by putative *Lmj*EXO1.

For this reason, we believe that two points must be considered. First, we cannot discard the existence of a subpopulation of promastigotes using alternative repair pathways to respond to genotoxic damage caused by IR. It is important to highlight, however, that the occurrence of HR-independent alternative DNA repair pathways does not exclude the possibility of ATR-promoted cell signaling. These two events could, in fact, happen simultaneously within the cell. Second, if the existence of ATR-promoted cell signaling in *L. major* is plausible, then an ATM-dependent signaling might also be, considering the important functions of this kinase in double-strand break responses in other organisms (Maréchal and Zou, 2013; Álvarez-Quilón et al., 2014; Rondeau et al., 2015).

In a previous study, our group demonstrated that the addition of specific inhibitors of human ATR and ATM rendered *L. major* wild-type promastigotes susceptible to hydrogen peroxide-induced damage in a concentration-dependent manner (Da Silva et al., 2018). We believed that these inhibitors would be proven useful to investigate the underlying mechanisms for the “EXO1” lineage hypersensitivity, owing to their higher specificity and efficacy when compared to caffeine. In subsequent susceptibility assays with hydrogen peroxide, we observed hypersensitivity of the putative *Lmj*EXO1 overexpressor *L. major* lineage to damage induced by the addition of an oxidative agent. These results were expected considering that the overexpression of putative *Lmj*EXO1 caused hypersensitivity to ionizing radiation, as discussed previously. However, our main goal was to investigate the effects of ATRi and ATMi on this lineage. Human EXO1 is phosphorylated by ATR in response to ionizing radiation and DNA replication inhibitors (El-Shemerly et al., 2008; Bolderson et al., 2009). In the present study, the addition of caffeine promoted a slight increase in the relative growth of the overexpressor lineage exposed to H_2_O_2_. This result is compatible with the faster recovery of “EXO1” lineage, after exposure to IR, when treated with caffeine. However, it is not clear how putative *Lmj*ATR and *Lmj*ATM modulate the response to oxidative damage in the parasite.

The contrasting phenotypes caused by the addition of ATRi and ATMi could help explain these mechanisms. We observed that the addition of ATRi promoted a higher sensitivity to H_2_O_2_ in the WT and pXG1 (control lineages). These lineages achieved 60% of the growth observed for the non-exposed to H_2_O_2_ counterparts, while the “EXO1” overexpressor lineage growth was reduced to 40%. Therefore, it is possible to affirm that the addition of ATRi promoted a phenotype similar to the overexpression of putative *Lmj*EXO1 in the control lineages, an effect that was also observed after the addition of caffeine to these controls exposed to IR. It has been demonstrated that VE-822 or siRNA-mediated depletion of ATR in HEK-293 cells results in EXO1 stabilization. It has been proposed that EXO1 degradation is a necessary event, mediated by ATR-dependent phosphorylation, in order to prevent hyper-resection of DNA ends (Tomimatsu et al., 2017). These findings could help support our first hypothesis, suggesting that the uncontrolled activity of putative *Lmj*EXO1 could lead to extended ssDNA filaments, a potentially deleterious event for *L. major* promastigotes.

We have proposed an investigation method for this hyper-resection promoted by the overexpression of putative *Lmj*EXO1, using 5-IdU detection by flow cytometry. We observed rapid and intense ssDNA formation 20 min after the addition of H_2_O_2_ to all analyzed *L. major* lineages. This ssDNA formation was increased in the putative *Lmj*EXO1 overexpressor lineage than in controls not treated with inhibitors, and it could be prolonged by the addition of ATRi to all *L. major* lineages. The rapid recruitment of EXO1 to damage sites is plausible, as has been reported in several other studies. For instance, EXO1 was shown to be recruited to double-strand breaks induced by laser micro-irradiation in MEF cells within 30 s post-lesion, in an EXO1-PIN domain-dependent manner (Zhang et al., 2015). Significant increases in EXO1 levels were also detected 30 min after damage induced by ionizing radiation in *T. brucei* promastigotes (Marin et al., 2018). These findings, when associated with the observation of larger ssDNA formation in ATRi-treated cells (especially in the overexpressor lineage), provide additional support for the hypothesis of a hyper-resection-dependent hypersensitivity of *L. major* promastigotes. In this case, we suggest that the longest ssDNA filaments could not be repaired appropriately and/or could lead to global genomic instability, in detriment of the parasite cell.

Contrary to ATRi, ATMi seems to exert a protective effect on the putative *Lmj*EXO1 overexpressor parasites, which reached growth rates similar to the ATMi-treated control lineages exposed to H_2_O_2_. In other organisms, ATM has been shown to act in the initial steps of homologous recombination, stimulating end resection, and phosphorylating and activating nucleases, including CtIP, MRE11, EXO1, and BLM. Consequently, ATM-deficient or ATM-inhibited cells exhibit a deficiency in the resection of DSB ends, as demonstrated by the reduced formation of RPA foci (Shiloh, 2003; Geuting et al., 2013; Shibata et al., 2014; Bakr et al., 2015). Taking this into consideration, we hypothesized that putative *Lmj*ATM could act at the beginning of DSB repair in *L. major*, promoting DNA resection by putative *Lmj*EXO1. This is further supported by the fact that the simultaneous addition of ATRi and ATMi did not alter the phenotype observed when ATMi was added alone. Moreover, when these findings are taken together, we suggest that caffeine, a non-selective inhibitor of these two protein kinases, acts in a similar way to the individual activity of ATMi. It is possible, therefore, that these two protein kinases might be acting at different times or processes of DNA repair in *L. major* exposed to oxidant agents, and the putative *Lmj*ATM could be recruited before end resection by *Lmj*EXO1, while putative *Lmj*ATR was subsequently recruited to these sites. In flow cytometry assays for the detection of 5-IdU foci, we observed that the addition of ATMi prevented ssDNA formation after exposure to H_2_O_2_ in all analyzed *L. major* lineages. This suggests that, when lacking *Lmj*ATM-dependent signaling, the enzyme *Lmj*EXO1, despite being overexpressed in the cell, could not be recruited to the lesion site. Therefore, this prevented the deleterious effects of hyper-resection, which could help explain the protective effect of ATMi in the overexpressor parasites.

**Figure 5:**
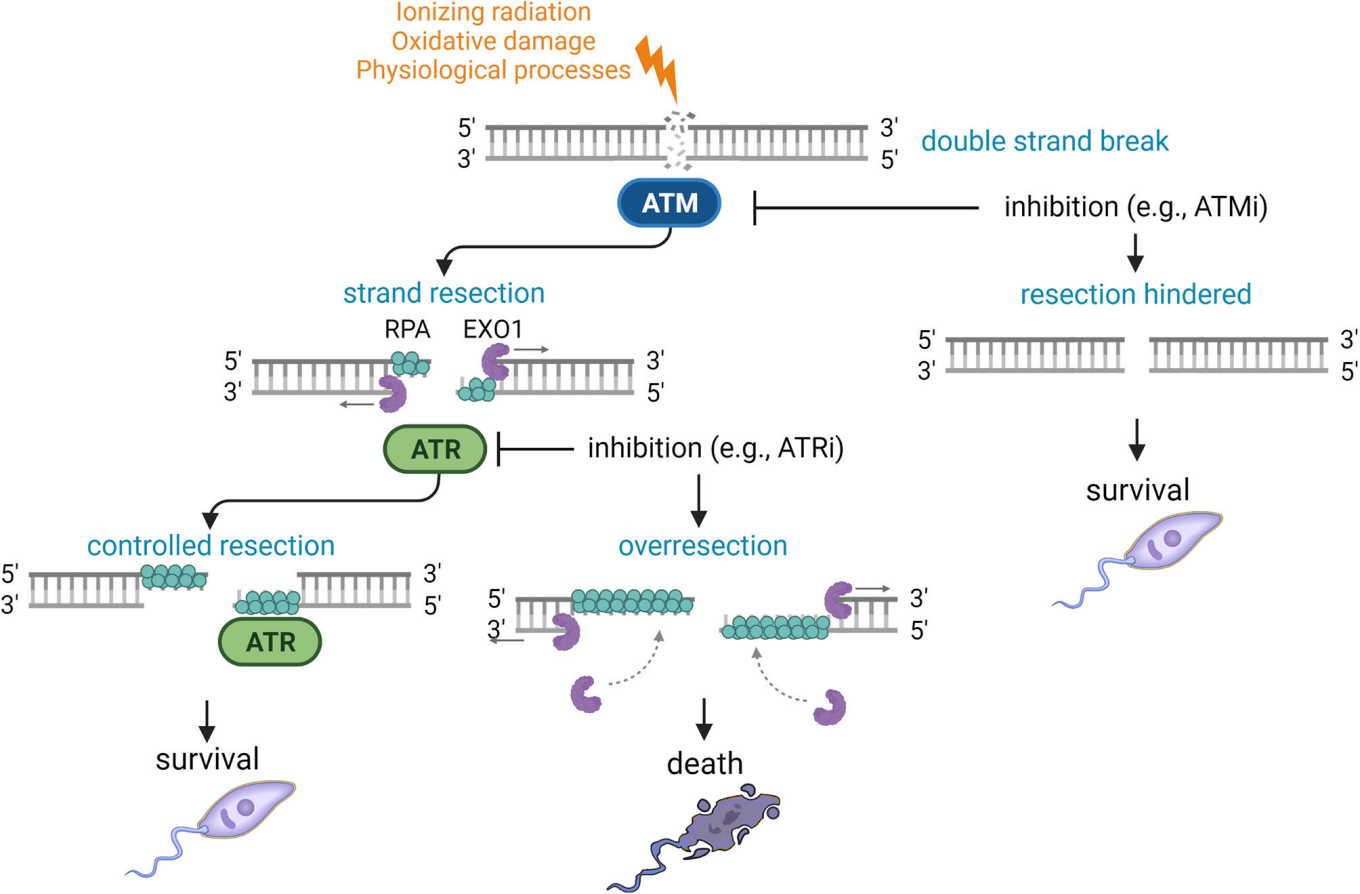
Theoretical model for the DNA damage and response in *Leishmania*. Schematic model to hypothesize the mechanisms controlling the DNA damage and repair in *Leishmania*, formulated according to the findings of the present work. Ionizing radiation, oxidative damage or endogenous physiological processes can cause many types of DNA damage (e.g. double strand breaks). In the damaged site, the putative homologue protein LmjATM (in blue) may be activated and stimulate the end resection by the enzyme LmjEXO1 (in purple). In other eukaryotes, many DNA damage responders, including the MRN-Sae2 complex, PCNA and RPA (in cyan), can activate EXO1. The extended 3’ ssDNA is covered by RPA, generating a signal for the recruitment of the putative homologue protein LmjATR (in green). As seen in other eukaryotes, LmjATR can modulate the end resection. After repairing the damage, the cell cycle can proceed and the parasite can recover and multiply. When overexpressed, or when the LmjATR modulation is deficient (e.g., by inhibition with ATRi), LmjEXO1 can continue the end resection, generation too long 3’ ssDNA ends, which can lead to generalized genomic instability and, consequently, to the parasite death. The parasites can possibly escape the deleterious effects of LmjEXO1 overexpression with the inhibition of ATM (e.g.: using ATMi), preventing the end resection. In this case, the parasites could use alternative pathways to repair the DNA damage. Lightning: DNA damage; grey arrow: end resection direction.

In other organisms, the end resection step commits the cell to the homologous recombination DNA repair pathway, because extended ssDNA filaments are inadequate substrates for binding of NHEJ factors (Symington and Gautier, 2011; Symington, 2016; Anand et al., 2018). It is plausible to believe that the addition of ATMi to *L. major* promastigotes exposed to H_2_O_2_ could have favored the activation of a resection-independent, alternative DNA repair pathway (**Figure 5**). This alternative pathway could help the parasite cope with the effects of the oxidant agent, independent of putative *Lmj*ATM/ATR signaling. In this discussion, we have already pointed out this possibility. We believe that a possible candidate for this alternative pathway could be microhomology-mediated end joining (MMEJ), an alternative form of end joining that takes place when NHEJ or homologous recombination fails, which has been previously demonstrated in *L. major* (Laffitte et al., 2016; Seol et al., 2018). Therefore, the investigation of these alternative pathways could help elucidate the mechanisms underlying the *Leishmania* response to genotoxic oxidative damage and the adaptation abilities of this resistant parasite to the adverse conditions it encounters throughout its life cycle.

## Data Availability Statement

The datasets presented in this study can be found in online repositories. The names of the repository/repositories and accession number(s) can be found below: https://tritrypdb.org/tritrypdb/app, LmjF.23.1270, https://www.ncbi.nlm.nih.gov/genbank/, NP_003677, http://www.wwpdb.org/, 3QE9, 5V04, 5V09, 5V0C, 5UZV, 1RXW, 3Q8M, 5KSE, 5K97, and 5UM9.

## Author Contributions

RBS, WRB, LLN, FBV, JDD, and AP performed the experiments. RBS, WRB, LROT, CRM, and ALP analyzed the data and statistics. RBS and ALP prepared the figures and tables. RBS and ALP wrote the manuscript. All authors reviewed the manuscript. All authors contributed to the article and approved the submitted version.

## Funding

This work was supported by grants from CNPq - Conselho Nacional de Desenvolvimento Científico e Tecnológico (grant number 408355/2016-6 - ALP) and FAPEMIG - Fundação de Amparo à Pesquisa do Estado de Minas Gerais (grant numbers APQ-00644-16 and PPM-00349-18 - ALP). RBS received a PhD fellowship from CAPES - Coordenação de Aperfeiçoamento de Pessoal de Nível Superior.

## Conflict of Interest

The authors declare that the research was conducted in the absence of any commercial or financial relationships that could be construed as a potential conflict of interest.

## Publisher’s Note

All claims expressed in this article are solely those of the authors and do not necessarily represent those of their affiliated organizations, or those of the publisher, the editors and the reviewers. Any product that may be evaluated in this article, or claim that may be made by its manufacturer, is not guaranteed or endorsed by the publisher.
