# Supplementary material for: Specific Human ATR and ATM Inhibitors Modulate Single Strand DNA Formation in Leishmania major Exposed to Oxidative Agent
Source: Front Cell Infect Microbiol. 2022 Jan 4;11:802613. doi: 10.3389/fcimb.2021.802613 (PMC8763966; doi:10.3389/fcimb.2021.802613)
Supplement: Supplementary file 1 [file DataSheet_1.zip › Data-Sheet_1/Exo1-ssDNA-Suppl-Material-Figures.pdf]

Supplementary Material

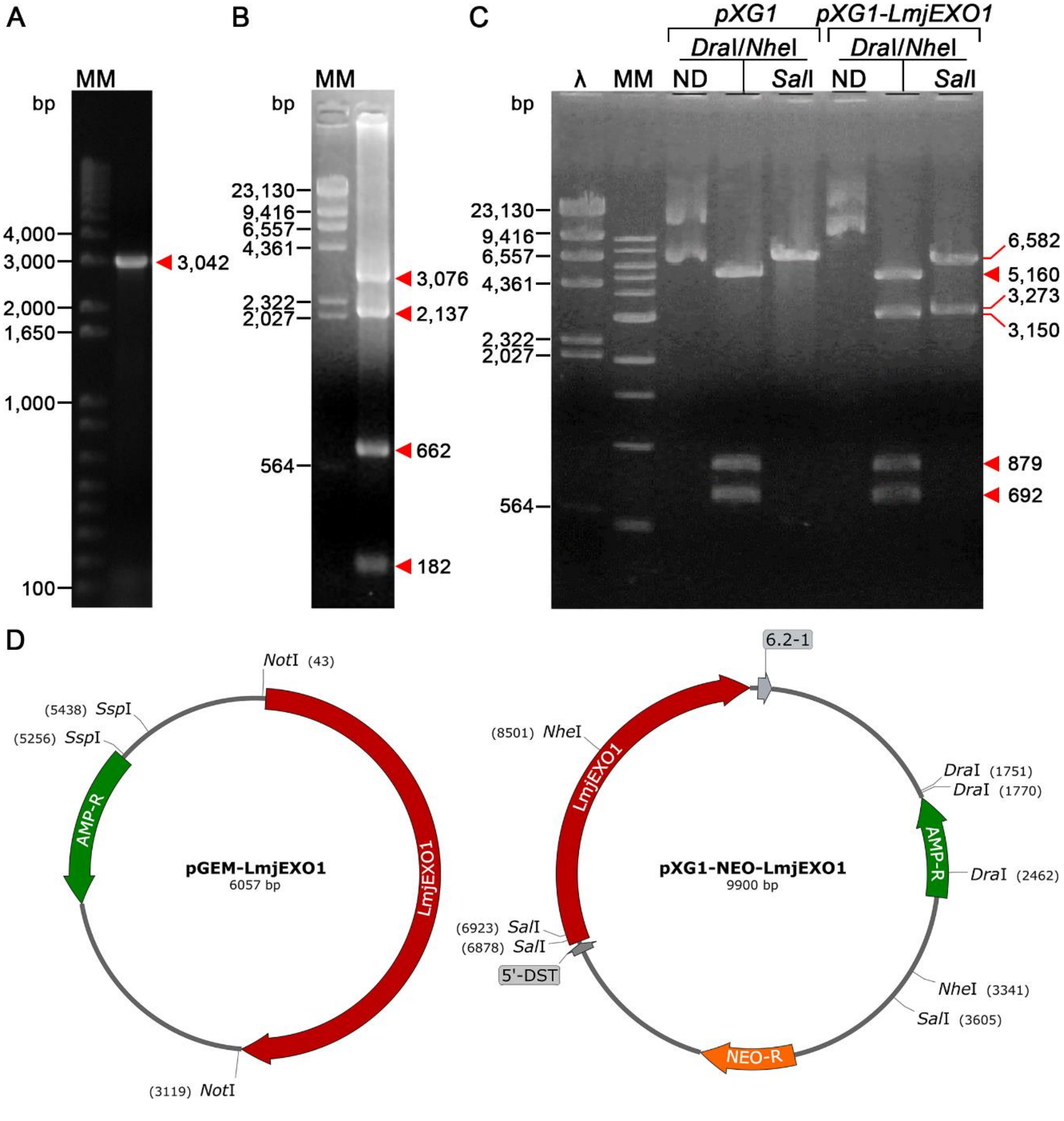

Supplementary Figure 1. Cloning of LmjEXO1 into *Leishmania* expression vector. **(A)** Putative *LmjEXO1* gene amplification using PCR. 0.8% (p/v) agarose gel stained with ethidium bromide showing the 3,042 bp amplification product, corresponding to the putative *LmjEXO1* gene. 1 kb Plus DNA Ladder was used as molecular marker (MM). **(B)** Digestion of pGEM *LmjEXO1* plasmid with nucleases *Not*I and *Ssp*I. 0.8% (p/v) agarose gel stained with ethidium bromide containing the nuclease digestion products with expected sizes of 3,076 bp (which contains the putative *LmjEXO1* insert); 2,137 bp; 662 bp and 182 bp. **(C)** Digestion of empty *pXG1 NEO* ("*pXG1"*) and *pXG1 NEO*

LmjEXO1 ("*pXG1-LmjEXO1*") plasmids with nucleases. 0.8% (p/v) agarose gel stained with ethidium bromide containing the products of single digestion with *Sal*I, double digestion with *Dra*I and *Nhe*I, or non-digested, coiled plasmids. The *Sal*I single cut of empty vector *pXG1 NEO* generated the expected 6,822 bp band, while the double cut of *pXG1 NEO LmjEXO1* rendered four bands: 5,160 bp; 3,150 bp; 879 bp and 692 bp. Remaining 19 bp band was lost during normal gel electrophoresis. The bands 5,232 bp, 879 bp and 692 bp were expected from *pXG1 NEO* digestion with DraI and NheI double digestion; a last 19 bp was off the gel. Digestion of *pXG1 NEO LmjEXO1* with DraI and NheI rendered the expected bands of 5,160 bp, 3,150 bp, 879 bp and 692 bp; a final 19 bp band was also lost during electrophoresis. **(D)** Schematic representation of the pGEM *LmjEXO1* (left) and *pXG1 NEO LmjEXO1* (right) plasmids, including the cleavage sites for nucleases. The arrows indicate the gene direction: *LmjEXO1* (*L. major* putative exonuclease-1 homologue – red), NEO^R (neomycin/G418 resistance gene – orange) and AMP^R (ampicillin resistance gene – green). The intergenic sequences that provide the signals for trans-splicing (5'-DST) and poliadenylation (6.2-1) (Ha et al, 1996) are represented as grey arrows.

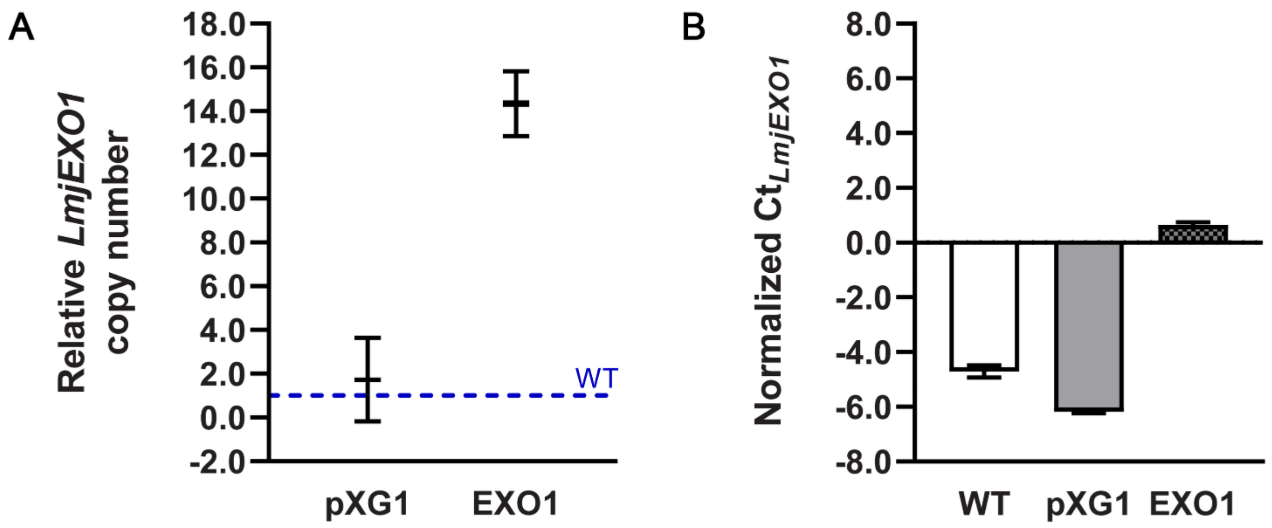

Supplementary Figure 2. Quantification of putative LmjEXO1 gene copy number and transcript levels. **(A)** Data shows mean ± standard deviation of the relative copy number of putative LmjEXO1 gene for episome vector control ("pXG1") and putative LmjEXO1 overexpressor lineage ("EXO1"), relative to wild-type ("WT"; dashed blue line). The relative copy numbers were calculated using Livak et al. (2001) method [49], considering the mean and standard deviation of the difference between the threshold cycle of analyzed lineage and wild-type Ct. **(B)** Data shows mean ± standard deviation of the difference between the threshold cycle obtained for the putative LmjEXO1 cDNA and the one obtained for the expression control gene, G6PD. Data from both graphs was obtained from triplicates of two independent experiments.

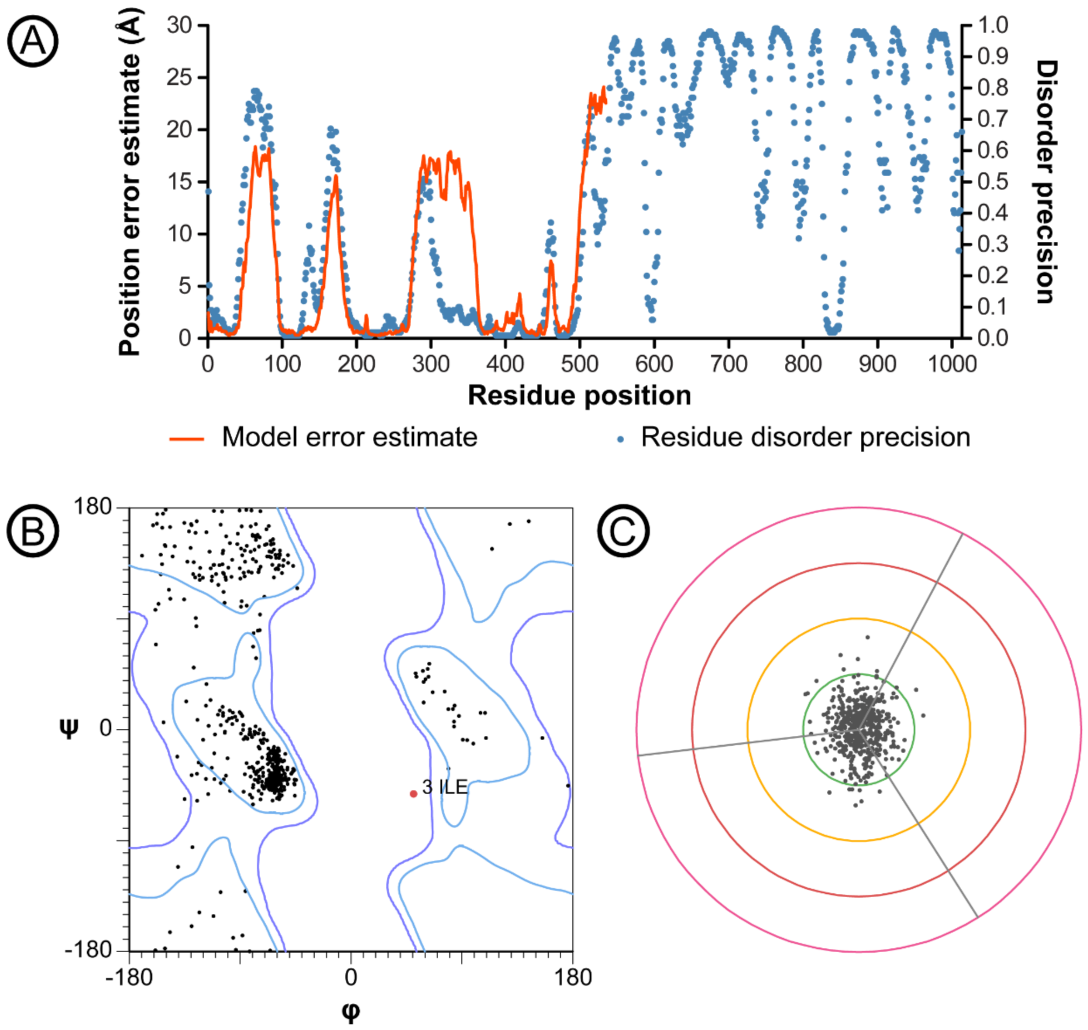

Supplementary Figure 3. Validation parameters of the predicted LmjEXO1 three-dimensional model. **(A)** The figure shows the estimate error, in angstroms (in orange), for the predicted position of each residue in the three-dimensional model of *Lmj*EXO1. It also displays the probability of intrinsic disorder of that residue (points in teal). The putative protein *Lmj*EXO1 presents a size of 1013 amino acids, which can be divided as a structured N-terminal region, including the first 535 residues, and a possibly disordered C-terminal region. The N-terminal region was used for the modelling of *Lmj*EXO1, using comparative modeling for the conserved sequences and *ab ignition* for the putative disordered sites. The last ones rendered the highest estimate errors for the position in the final model. The validation parameters for the model were obtained using MolProbity (http://molprobity.biochem.duke.edu/). **(B)** Ramachandran map for *Lmj*EXO1 model. Each data point indicate the dihedral angles psi (ψ) and phi (φ) of each residue. Residues in favorable regions are inscribed in the light blue lines, whereas the residues in allowed regions are inscribed in the dark blue lines. Only one isoleucine residue (residue number 3) was found in not allowed region (red point). **(C)** Carbon beta deviation map for *Lmj*EXO1 three-dimensional model. The closer the points are to the center, the lower the deviation is. Residues with a deviation > 0,25 Å (yellow circle) are considered not allowed outliers.

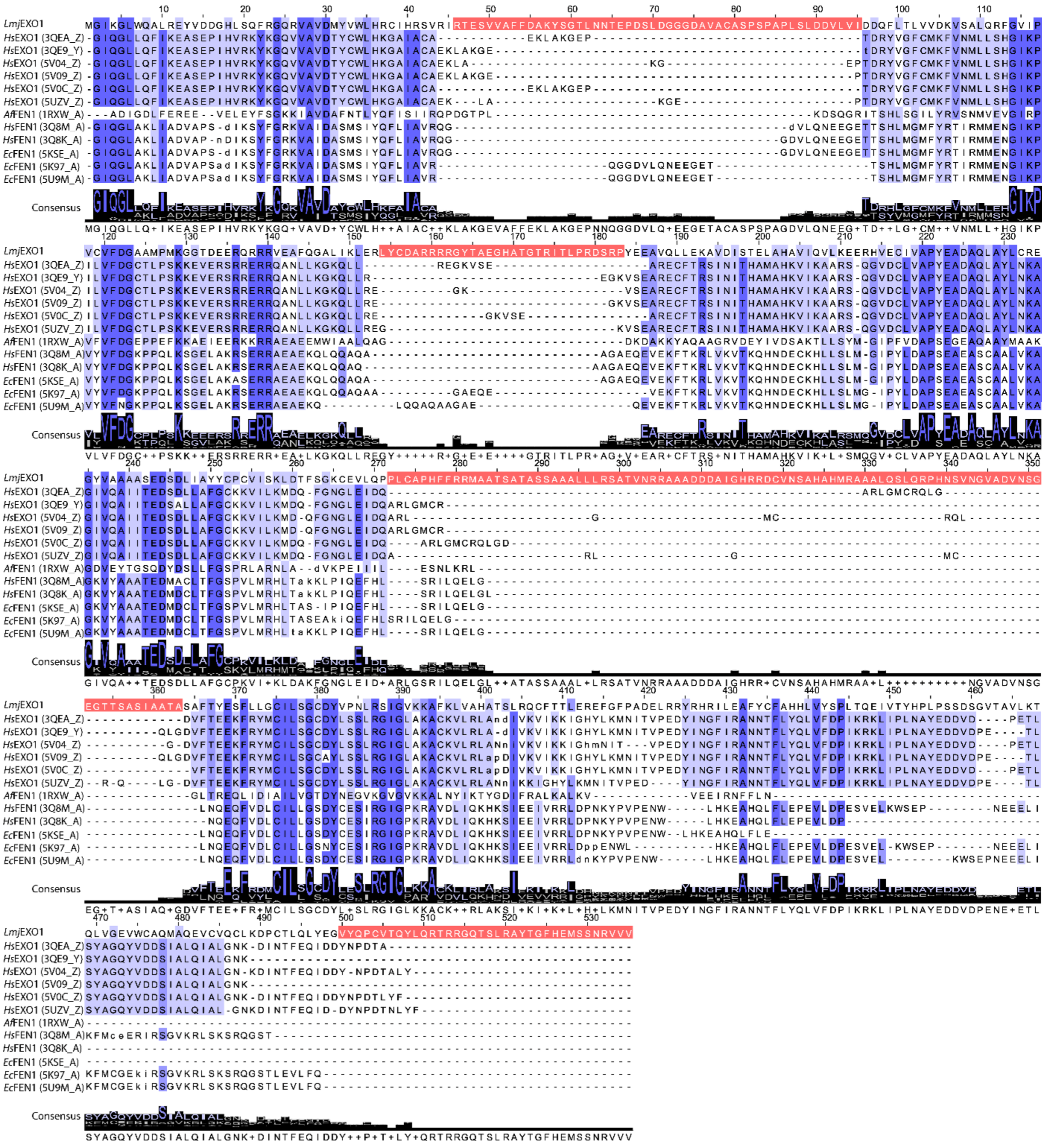

Supplementary Figure 4. Template alignment for the construction of LmjEXO1 three-dimensional model. The figure shows the alignment used for comparative modeling of the putative enzyme EXO1 of L. major (LmjEXO1). Amino acid residues highlighted in blue represent their conservation among the sequences: the darker the shade, the greater its conservation. Residues highlighted in red represent insertion segments in LmjEXO1, which were then modeled by ab initio. The histogram below the sequences represent the amino acid consensus for each position. The codes in brackets are the accession numbers for the sequences in the PDB databank. HsEXO1: human EXO1 (Homo sapiens); AfFEN1: Archaeoglobus fulgidus FEN1; HsFEN1: human FEN1; EcFEN1: Escherichia coli FEN1.

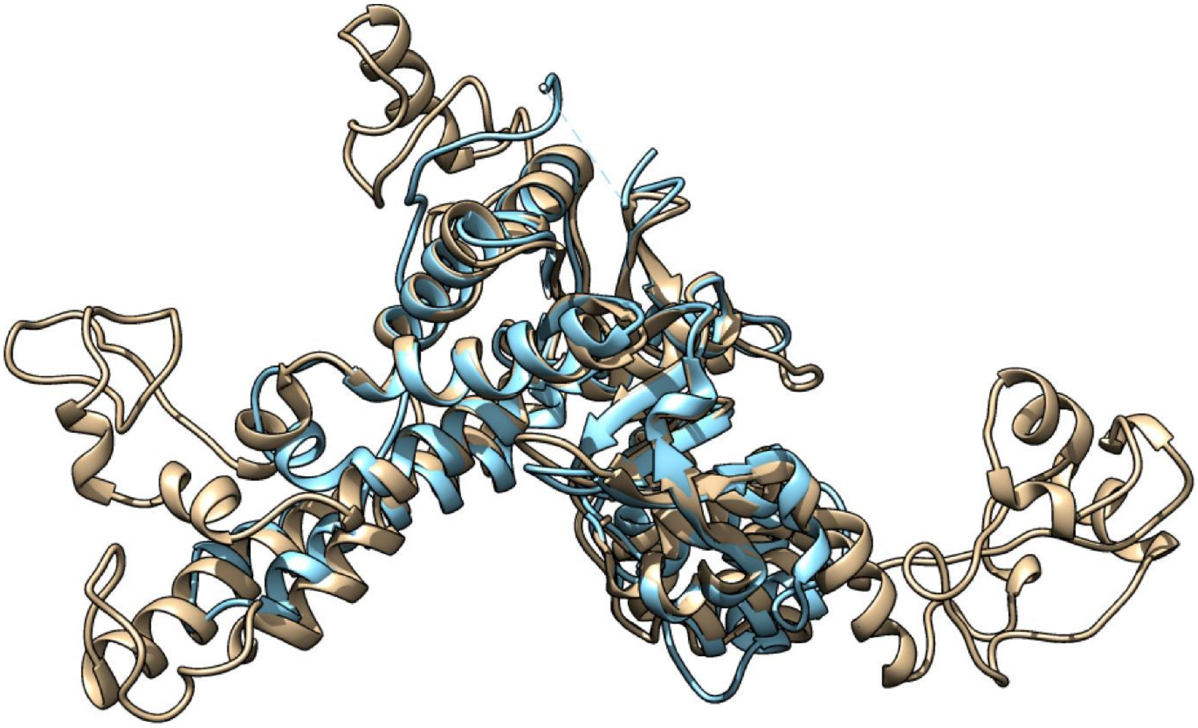

Supplementary Figure 5. Structural alignment of HsEXO1 and the predicted model of LmjEXO1. The figure shows the structural overlap of predicted model for *Lmj*EXO1 (in beige), and the available *Hs*EXO1 experimental structure (in blue; PDB ID 5V06 (Shi et al, 2017)) for comparison. It is possible to observe the alignment of the main domains, which are conserved in the parasite protein. Conversely, the not aligned stretches, which were predicted to be disordered regions, were modeled using *ab initio* strategy. The structural alignment was visualized using Chimera v. 1.11.2.

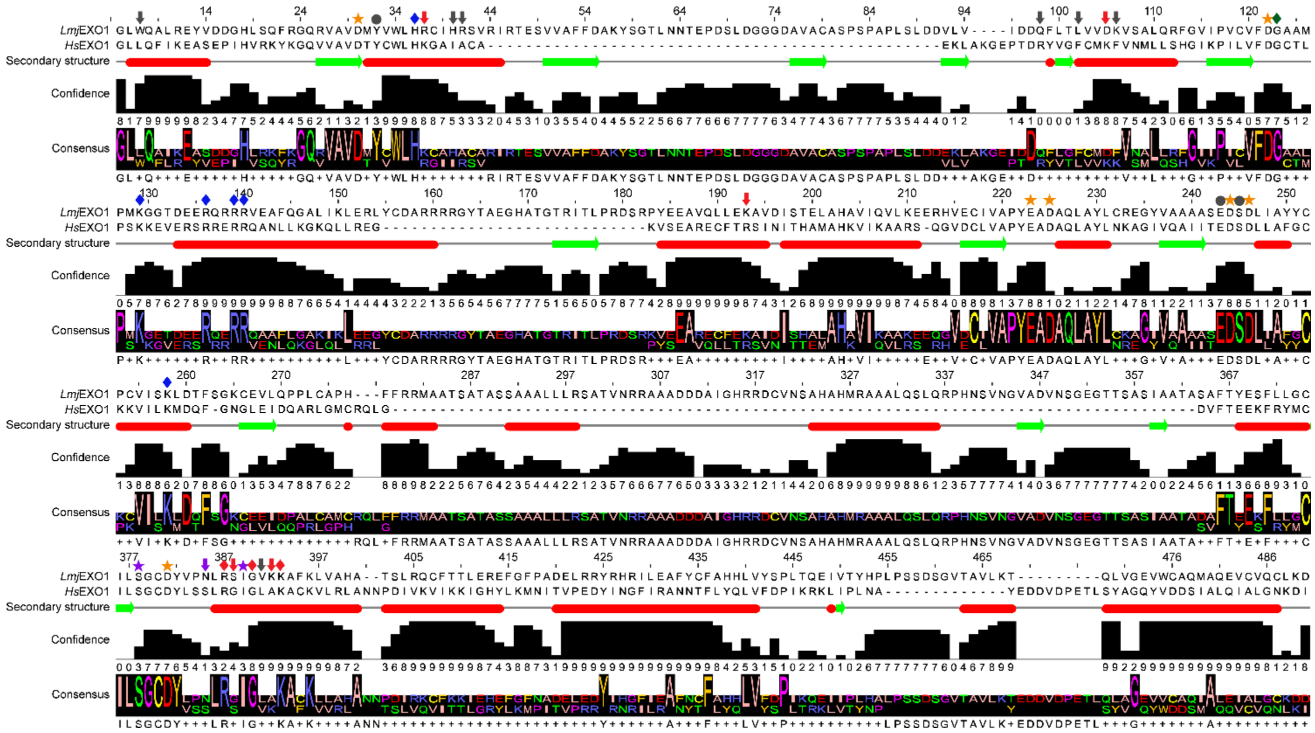

Supplementary Figure 6. Structural conservation of important residues in the putative protein LmjEXO1. Sequence alignment map for the proteins HsEXO1 and putative LmjEXO1, according to their three-dimensional alignment (Supplementary Figure 4). Secondary strucutures are represented as red bars for α-helixes and green arrows for β-strands; gaps in the alignment are represented as dashes (-); more than one possible residue for the consensus is represented as a plus signal (+). Orange stars: conserved active site residues; purple stars: conserved K+ chelating residues; purple arrows: substituted K+ chelating residues; blue diamonds: conserved residues for the interaction with complementary (non substrate) DNA strand; red diamonds: conserved residues for the interaction with substrate DNA strand; red arrows: substitutions of these residues; grey circles: conserved residues for the Van der Waals interactions with DNA; grey arrows: substitutions of these residues; green diamond: conserved glycine residue acting as hinge for the mobile arc (α4-α5 microdomain). The numbers above the sequence indicate the position of that residue in the L. major protein.

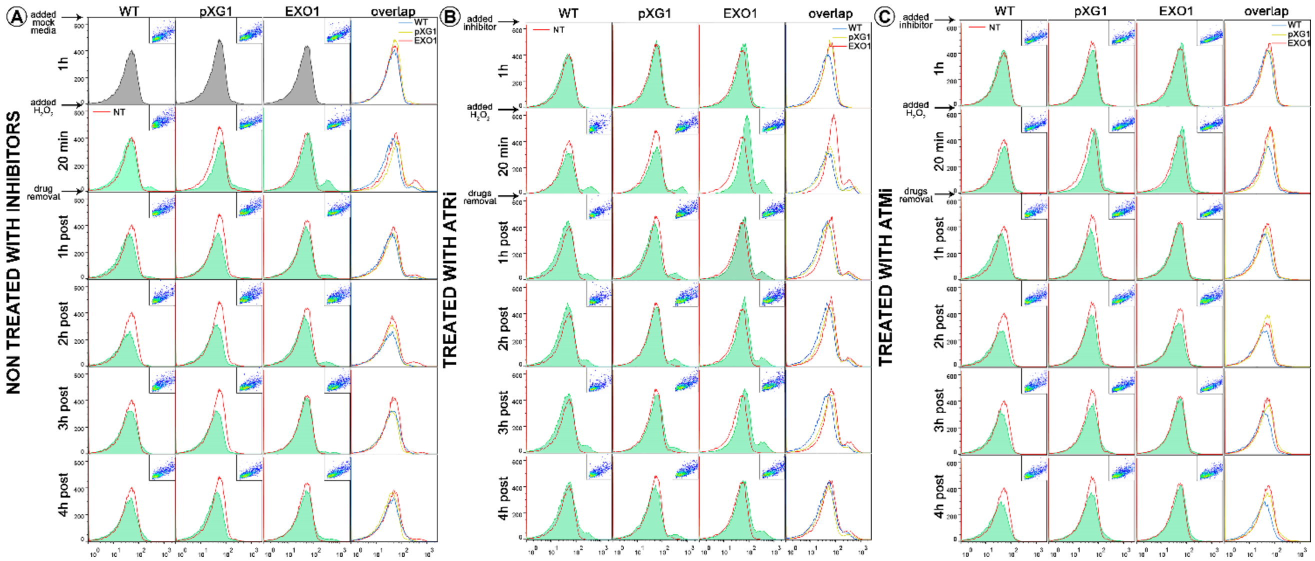

Supplementary Figure 7. Histograms of the ssDNA formation analysis in *L. major* lineages exposed to H_2O_2. The histograms compare the ssDNA formation kinetics in not treated and not exposed to H2O2 cells (red line histograms), with cells exposed to H2O2 (green shaded histograms). The data was plotted in the graphs of Figure 4B. L. major promastigotes were incubated during 1 hour in standard culture media **(A)** or media containing 10μM ATRi 10μM **(B)** or 10μM ATMi **(C)**. Then, the parasites were exposed to 500 μM H2O2 during 20 minutes. The parasites were washed for complete removal of the drugs and resuspended in standard culture media. 0,5 to 2×107 parasites samples were collected 1 hour after the addition of inhibitors (starting points of the curves), after 20 minutes of the addition of H2O2, and then every hour after the removal of the drugs, until 4 hours post exposure to H2O2. After fixation, the parasites were incubated with anti-BrdU FITC conjugate for the detection of 5-IdU nucleotide. The forth column of histograms show the overlap of the wild type (WT, blue line), episome vector control (pXG1, golden line) and LmjEXO1 overexpressor lineage (EXO1, red line) histograms of each row. The population of parasites analyzed to compose each histogram is shown by the insets in the upper right corners. A fluorescence intensity of 102 UA was considered the threshold for this assay, representing ssDNA formation above the normal.
