# Supplementary material for: Specific Human ATR and ATM Inhibitors Modulate Single Strand DNA Formation in Leishmania major Exposed to Oxidative Agent
Source: Front Cell Infect Microbiol. 2022 Jan 4;11:802613. doi: 10.3389/fcimb.2021.802613 (PMC8763966; doi:10.3389/fcimb.2021.802613)
Supplement: Supplementary file 1 [file DataSheet_1.zip › Data-Sheet_1/Exo1-ssDNA-Suppl-Material-Table-1.pdf]

Supplementary Table 1. List of primers used in the present work.

Primer name	Sequence (5' to 3')	Tm*	Product size (bp)	Efficiency	Purpose
LmjEXO1-F1	ATGGGAATCAAAGGTCTCTG	52.0	3,042	NA**	*LmEXO1*
LmjEXO1-R1	CTATGTCTTCTTGAACAACATC	49.2			cloning
LmjEXO1-PW1-F	The same as LmjEXO1-F1	52.0	1,980	NA**	Insert
LmjEXO1-PW1-R	GTGCGCCAACGAAGCAAA	57.0			sequencing
LmjEXO1-PW2-F	CATCACGCTTCCGAGAGACT	56.7	1,997	NA**	by primer
LmjEXO1-PW2-R	CACGGTTGCACTGCCAATAG	56.8			walking
LmjEXO1-PW3-F	CAGCGACCACATAACTCCGT	57.2	2,037	NA**	
LmjEXO1-PW3-R	The same as LmjEXO1-R1	49.2			
LmjEXO1-QF	GCACTGCTGTGTGCGAGCATTGGT	68.0	90	90%	qPCR
LmjEXO1-QR	GCACCAACATGTGCACCCCCGA	68.3			
LmjG6PD-QF	ACCGCATTGACCACTACCTC	62.0	102	96%	
LmjG6PD-QR	GATGTTGTTCGAGTTCCAC	56.0			

*Tm: melting temperature in °C.

**NA: Not applicable.
