# Supplementary material for: Specific Human ATR and ATM Inhibitors Modulate Single Strand DNA Formation in Leishmania major Exposed to Oxidative Agent
Source: Front Cell Infect Microbiol. 2022 Jan 4;11:802613. doi: 10.3389/fcimb.2021.802613 (PMC8763966; doi:10.3389/fcimb.2021.802613)
Supplement: Supplementary file 1 [file DataSheet_1.zip › Data-Sheet_1/Exo1-ssDNA-Suppl-Material-Table-2.pdf]

Supplementary Table 2. Percentage of *L. major* promastigotes at each stage of the cell cycle after exposure to ionizing radiation.

Day	Lineage	Cell cycle stage					4N/2N ratio	Total cells analyzed	p value[a]
		<G1	G1	S	G2	>G2			
0	WT	18,82	44,48	8,01	25,65	26,84	1,99	11757	0,479
	pXG1	20,78	35,63	15,01	20,48	8,10	2,04	11575	
	EXO1	17,06	39,32	9,21	25,94	8,47	2,05	11914	
1	WT	21,28	44,27	11,56	20,6	21,13	1,99	12981	0,447
	pXG1	16,59	43,41	7,56	25,72	6,72	2,04	11929	
	EXO1	15,84	38,26	10,75	24,95	10,2	1,95	12104	
2	WT	16,99	48,03	11,09	20,42	29,49	2,02	12260	0,424
	pXG1	18,03	40,11	8,06	25,73	8,06	1,95	12023	
	EXO1	14,32	42,22	7,69	23,33	12,44	1,95	14511	
3	WT	17,69	43,07	12,22	22,01	29,82	2,03	13407	0,336
	pXG1	16,63	38,25	7,19	30,12	7,8	1,95	12542	
	EXO1	15,21	37,66	6,41	26,27	14,46	1,95	13203	
4	WT	16,02	43,46	9,79	26,71	34,04	2,05	13084	0,184
	pXG1	15,23	43,15	6,84	23,81	10,97	2,02	13533	
	EXO1	19,67	29,23	8,06	27,98	15,06	1,96	14339	
5	WT	21,44	40,99	9,62	22,29	30,79	1,99	13373	0,177
	pXG1	15,44	33,6	10,74	27,58	12,65	1,95	12972	
	EXO1	23,38	33,14	2,76	29,85	10,87	1,95	14798	
6	WT	25,24	43,76	10,84	17,39	30,37	2,08	12666	0,227
	pXG1	22,05	37,46	9,79	21,53	9,17	1,99	12231	
	EXO1	23,34	29,87	8,1	27,6	11,1	1,95	14195	
7	WT	20,57	40,61	10,51	25,17	26,16	1,99	11570	0,852
	pXG1	15,7	39,8	11,14	27,9	5,47	1,98	11659	
	EXO1	**	**	**	**	**	**	**	
8	WT	14,86	55,25	10,79	15,88	24,89	2,06	12488	<0,0005
	pXG1	18,2	42,48	9,19	25,13	5	2,01	11760	
	EXO1	42,91	29,13	10,79	9,96	7,21	2	16711	
9	WT	19,97	64,67	4,23	10,78	11,99	2,14	12085	<0,0005
	pXG1	7,7	57,33	7,5	22,83	4,64	2,11	11019	
	EXO1	27,13	34,47	8,95	22,94	6,51	2,14	13343	
